# Dexmedetomidine Preserves Hippocampal Neurogenesis During Recovery from Neonatal Hyperoxia in Rats

**DOI:** 10.3390/cells15121094

**Published:** 2026-06-16

**Authors:** Stefanie Endesfelder, Christoph Bührer, Thomas Schmitz

**Affiliations:** 1 Department of Neonatology, Charité—University Medical Center Berlin, Augustenburger Platz 1, 13353 Berlin, Germany; christoph.buehrer@charite.de; 2 Clinic for Pediatrics and Adolescent Medicine, University Hospital of the Brandenburg Medical School, Fehrbelliner Straße 38, 16816 Neuruppin, Germany; t.schmitz@ukrb.de

**Keywords:** neonatal hyperoxia, dexmedetomidine, hippocampal neurogenesis, oxidative stress, neurodevelopmental dysmaturation, apoptosis, autophagy

## Abstract

Neonatal hyperoxia induces oxidative stress that disrupts neurodevelopmental processes. While dexmedetomidine (DEX) exhibits acute neuroprotective properties, its long-term impact on developmental trajectories during recovery remains incompletely understood. This study examined whether a single neonatal dose of DEX modulates hippocampal neurogenesis following hyperoxia across defined postnatal stages. Six-day-old *Wistar* rats were exposed to 80% oxygen for 24 h and evaluated at postnatal days (P) 9, 11, and 14 after recovery in room air. Mechanistically, hyperoxia permanently triggered apoptotic cascades, evidenced by sustained transcript upregulation and increased histological apoptosis and cell loss across the cortex and hippocampus, while disrupting the hippocampal progenitor niche, suppressing key differentiation factors (*Sox2*, *Tbr2*, *Prox1*, *Calb1*) and altering mature NeuN expression. Likewise, markers for autophagy (Atg5/12, Beclin1), neurotrophins (*BDNF*, *NGF*, *NT3*), and plasticity markers (*Nrp1*, *Sem3a*) showed reduced expression. Proactive treatment with DEX (5 µg/kg) significantly reversed these detrimental patterns. First, DEX elicited a robust antioxidant response (*Nrf2*, *SOD1*, *SOD3* induction). Second, DEX effectively suppressed hyperoxia-induced programmed cell death and tissue degeneration up to P14. Crucially, this dual protection sustained the neurogenic niche, safeguarding autophagy processes as well as neurotrophic and neuronal plasticity mediators, while showing excellent safety under normoxia. In conclusion, a single dose of DEX mitigates acute oxygen injury and exhibits beneficial, stage-specific effects within hippocampal neurogenic niches during the postnatal phase, highlighting its potential to preserve neurodevelopmental trajectories.

## 1. Introduction

The transition from the hypoxic intrauterine environment to atmospheric hyperoxia represents a significant physiological challenge for preterm infants [1,2]. Due to immature antioxidant defense systems and underdeveloped endogenous radical scavenger mechanisms, even brief periods of high oxygen exposure can trigger prolonged oxidative stress [3,4]. This “oxygen paradox” is a primary driver of neonatal brain injury, as the developing central nervous system is exquisitely vulnerable to oxidative toxicity [5,6,7].

Hyperoxia induces the overproduction of reactive oxygen species (ROS), leading to cellular damage via lipid peroxidation as well as DNA and protein oxidation [8,9]. These molecular insults culminate in widespread programmed cell death, including the apoptosis of neurons and glial cells across both white and grey matter [10,11,12]. Beyond immediate cell loss, hyperoxia severely disrupts critical developmental programs. It impairs oligodendrocyte maturation, leading to myelination deficits and structural white matter anomalies, while simultaneously disturbing neural progenitor cell populations and cerebrovascular development [13,14,15]. Furthermore, the activation of pro-inflammatory cytokines exacerbates this damage, creating a neuroinflammatory milieu that compounds the initial injury [14,16]. Clinical evidence underscores this vulnerability, associating neonatal hyperoxia with long-term motor, cognitive, and behavioral deficits [14,17].

The neonatal period is defined by a rapid convergence of fundamental processes, including neurogenesis, synaptogenesis, and the proliferation of oligodendrocyte progenitor cells [18]. In the last third of pregnancy, the human brain undergoes a fourfold increase in volume, characterized by intense dendritic arborization and cortical folding [19]. In this context, injury does not merely cause a loss of function but triggers “dysmaturation”, a fundamental decoupling of the brain’s developmental trajectory. For instance, hyperoxia-induced oxidative stress causes a maturation arrest in pre-oligodendrocytes by disrupting essential transcription factors [17,20,21,22]. Furthermore, both hippocampal and cerebellar neurogenesis can be significantly impaired and delayed [17,23,24,25,26,27,28]. Since brain development is hierarchical, these early injuries can lead to maladaptive ontogenetic adjustments, i.e., short-term restructuring for survival, which can then result in long-term cognitive dysfunctions [22,29,30].

In the clinical setting, the management of preterm infants often requires sedation; however, traditional agents like opioids and benzodiazepines have been linked to poor neurological outcomes [31]. Midazolam, in particular, is associated with hippocampal dysmaturation and reduced hippocampal volume [32,33,34]. Consequently, there is an urgent need for sedative alternatives that offer neuroprotective properties. Dexmedetomidine, a highly selective alpha2-adrenoceptor agonist, has emerged as a promising candidate. Beyond its sedative effects, dexmedetomidine exerts multi-modal neuroprotection by inhibiting NADPH oxidase 2 (NOX2) to reduce ROS production, suppressing microglial activation, and promoting cell survival through the PI3K/Akt signaling pathway [35,36]. Furthermore, it has been shown to protect synaptic integrity and restore the expression of markers such as synaptophysin following hypoxic–ischemic insults [35]. Recent clinical data demonstrate that dexmedetomidine reduces cumulative opioid exposure, shortens the time to enteral feeding, and significantly lowers seizure risk (OR: 0.31) in neonates with hypoxic–ischemic encephalopathy [37,38]. Mechanistically, these benefits are driven by dexmedetomidine’s capacity to suppress oxidative stress and inflammation, while preserving mitochondrial function and preventing apoptosis [39].

Despite these promising findings, important gaps remain in our understanding of dexmedetomidine’s efficacy. While data in cerebellar Purkinje cells in neonatal mice indicated improved cell survival beyond the second postnatal week after a single dexmedetomidine administration [26], most preclinical studies have focused on acute molecular protection, representing snapshot analysis immediately after acute hyperoxic damage within the first week of life in rodents [40,41,42,43]. While insights from these studies confirm that a single dose of dexmedetomidine may reduce expression of markers for neurodegeneration and inflammation, they do not account for the developmental dynamic during brain growth spurt of the immature brain. It remains unclear whether dexmedetomidine administration causes a sustained preservation of the developmental trajectory or whether it only coincides with short-term modifications in the injured brain. Specifically, the long-term impact on progenitor pool dynamics, the essential transition from excitatory to inhibitory GABAergic signaling, and the modulation of autophagy and neurotrophic factors like BDNF across multiple postnatal stages have not been elucidated.

The aim of the present study is to shift the neuroprotective paradigm from acute damage modification to the long-term maintenance of neurodevelopmental programs. By utilizing a serial investigation approach across multiple postnatal time points (P9, P11, and P14), we aim to clarify whether dexmedetomidine maintains the coordinated maturation of the hippocampus under a short phase of hyperoxic conditions during the vulnerable phase of brain development. We hypothesize that dexmedetomidine treatment mitigates acute oxidative injury and sustains the integrity of neural progenitor niches and synaptic plasticity, thereby preserving functional and structural development of the neonatal brain.

## 2. Materials and Methods

### 2.1. Animal Welfare

Time-mated Wistar rats (Janvier Labs, Le Genest-Saint-Isle, France) were housed individually under standardized conditions, including a 12 h/12 h light–dark cycle, constant room temperature, and 60% relative humidity. The animals had ad libitum access to water and a standard diet (Altromin 1324, Lage, Germany; containing 24% protein, 11% fat, and 65% carboHYDEXrates). In all experiments, neonatal pups remained with a lactating dam throughout the study period. All animal procedures were reviewed and approved by the local regulatory authority (LAGeSo, approval number G-0145/13 on 1 July 2013, and G-0139/18 on 3 July 2018) and were conducted in accordance with institutional regulations and ARRIVE guidelines (Animal Research: Reporting of In Vivo Experiments), as detailed by Percie du Sert et al. [44].

### 2.2. Oxygen Exposure and Drug Administration

As described previously [26,27,40,41], pups were randomized by litter and sex at postnatal day (P) 4. Subsequently, dams with their litters were exposed to either hyperoxic conditions (80% O_2_; OxyCycler BioSpherix, Lacona, NY, USA) or normoxic conditions (21% O_2_) from P6 to P7. Dexmedetomidine (DEX; 5 µg/kg body weight; Dexdor^®^, Orion Pharma, Espoo, Finland), diluted in phosphate-buffered saline (PBS), was administered intraperitoneally (i.p.; 100 µL/10 g body weight). Treatment (DEX or vehicle [0.9% saline]) was given once, 15 min prior to oxygen exposure. Animals were allocated to one control group (NO: 21% O_2_) and three experimental groups (NOD: 21% O_2_ + 5 µg/kg DEX; HY: 80% O_2_ + 0.9% saline; HYDEX: 80% O_2_ + 5 µg/kg DEX), with six pups per group. The dose of 5 µg/kg dexmedetomidine (DEX) was chosen based on comprehensive prior dose–response studies in this specific neonatal hyperoxia [40,41]. Previous evaluations comparing 1, 5, and 10 µg/kg DEX established that 5 µg/kg represents the optimal neuroprotective concentration. Following oxygen exposure at P7, all animals were maintained under normoxic room air conditions and remained with a lactating dam until the respective analysis time points at P9, P11, and P14, when assessments were performed. All experimental procedures followed previously established protocols [26,27,40,41].

### 2.3. Tissue Preparation

At P9, P11, and P14, pups were anesthetized via intraperitoneal injection of ketamine (100 mg/kg), xylazine (20 mg/kg), and acepromazine (3 mg/kg), followed by transcardial perfusion as previously described [26,27]. After decapitation and cranial opening, the entire brain was rapidly excised. For gene expression analyses, tissues were perfused with PBS (pH 7.4), snap-frozen in liquid nitrogen, and stored at −80 °C. For immunohistochemical analyses, perfusion was performed with PBS (pH 7.4) followed by 4% paraformaldehyde (PFA, pH 7.4). Samples were post-fixed in PFA at 4 °C for 24 h, dehydrated through a graded ethanol series, and embedded in paraffin.

For subsequent histological analyses, the anatomical identification and quantitative analysis of the frontal cortex (FC), retrosplenial cortex (RSC), and other brain regions were performed in situ on intact coronal brain sections using a standardized neonatal rat brain atlas. Serial sections were consistently sampled at defined stereotaxic coordinates relative to Bregma. This atlas-guided approach enabled reliable delineation of the respective cortical regions and ensured that cellular quantification was confined to the anatomically defined areas of interest.

### 2.4. RNA Extraction and Quantitative Real−Time PCR

As described previously [26,27,40,41], total RNA was isolated from whole snap-frozen cerebral tissue using peqGOLD RNAPure™ (PEQLAB Biotechnologie, Erlangen, Germany) according to the manufacturer’s protocol. Following DNase treatment, 2 µg of RNA was reverse-transcribed into cDNA.

Quantitative real-time PCR was conducted using qPCR BIO Mix Hi-ROX (NIPPON Genetics Europe, Düren, Germany). Target gene expression (Supplementary Table S1) was measured with the QuantStudio™ 5 Real-Time PCR System (Applied Biosystems, Thermo Fisher Scientific Inc., Waltham, MA, USA). Fluorescent reporter and quencher-labeled probes were used for detection. All samples were analyzed in technical triplicates, and mean Ct values were used for calculation. Relative expression levels were calculated using the 2^−ΔΔCt^ method [45]. Gene expression levels were normalized to hypoxanthine–guanine phosphoribosyltransferase (HPRT) as the internal reference. The underlying numerical 2^−ΔΔCt^ datasets for the respective figures are provided in Supplementary Tables S2–S4.

### 2.5. Immunohistochemistry

Paraffin-embedded cerebral tissue was sectioned at 5 µm, mounted on SuperFrost Plus slides, deparaffinized in xylene substitute (ROTI^®^Histol, Carl Roth, Karlsruhe, Germany, Cat. 6640), and rehydrated through graded ethanol. Heat-induced antigen retrieval was performed in citrate buffer (pH 6.0) in a microwave oven for 10 min at 600 W; for cleaved caspase-3 (cCasp3), this was followed by additional pretreatment with 3% H_2_O_2_ and intermediate washes in PBS and distilled water. Non-specific binding was blocked for 1 h at room temperature using antibody-specific blocking solutions (for Pax6/PCNA and cCasp3: 2% (*v*/*v*) goat serum, 1% (*w*/*v*) BSA, 0.1% (*v*/*v*) Triton X-100, 0.05% (*v*/*v*) Tween-20, 0.1% (*w*/*v*) gelatin in PBS; for Calb1 and NeuN: 3% (*w*/*v*) BSA, 0.2% (*v*/*v*) Triton X-100 in PBS). Sections were then incubated overnight at 4 °C with the respective primary antibodies diluted in antibody diluent (Zymed Laboratories, San Francisco, CA, USA, Cat. ZUC103): rabbit anti-PAX6 (GeneTex Europe GmbH, Freising, Germany, Cat. GTX113241, 1:250) together with mouse anti-PCNA (Abcam, Cambridge, UK, Cat. ab29, 1:500), mouse anti-Calbindin (Abcam, Cat. ab75524, 1:500), mouse anti-NeuN (Merck Millipore, Darmstadt, Germany, MAB377, 1:200), or rabbit anti-cleaved caspase-3 (Cell Signal Technology, Danvers, MA, USA, Cat. 9661, 1:400). After PBS washing, appropriate fluorophore-conjugated secondary antibodies were applied for 1 h at room temperature (goat anti-mouse Alexa Fluor 488, Thermo Fisher Scientific, Schwerte, Germany, Cat. A11001, 1:200; goat anti-rabbit Alexa Fluor 594, Thermo Fisher Scientific, Cat. A11037, 1:200; or goat anti-rabbit Alexa Fluor 488, Thermo Fisher Scientific, Cat. A11034, 1:200), followed by nuclear counterstaining with DAPI (Sigma-Aldrich, Taufkirchen, Germany, Cat. D9542, 1:1000 in PBS for 10 min). Finally, sections were washed in PBS and coverslipped with Immu-Mount (Thermo Fisher Scientific, Cat. 9990412). For all staining, species- and isotype-matched IgG controls were processed in parallel under identical conditions to verify specificity of the immunolabeling.

### 2.6. TUNEL Staining

As described previously [41], paraffin-embedded sections were cut (5 μm), deparaffinized in ROTI^®^Histol twice for 10 min each, rehydrated in descending ethanol series, and rinsed in phosphate-buffered saline for 3 min each at room temperature. After deparaffinization of sections, an in situ detection of cells with DNA-strand breaks was performed by the TUNEL labeling method using a TdT-FragEL DNA fragmentation detection kit (Merck Millipore) according to the manufacturer’s instructions. Negative controls were performed by substituting Tris-buffered saline for the TdT enzyme.

### 2.7. Image Acquisition and Quantification

#### 2.7.1. Fluorescence Imaging

For qualitative analyses and quantification of PAX6/PCNA co-labeling, single Calb1 and NeuN staining, and cleaved caspase-3 (cCasp3), sections were digitized using a Keyence fluorescence microscope (BX800) equipped with BZ-II Viewer (version 01.03.00.01) and BZ-II Analyzer (version 1.1.2.4) software. Images were acquired with a 10× objective, and individual RGB channels were automatically merged before analysis. All images for a given staining, time point, and brain region were captured in a single session using identical exposure times and contrast/brightness settings across all experimental groups. Quantitative analyses were performed blinded to group allocation.

For PAX6/PCNA/DAPI staining, the dentate gyrus (DG) was evaluated with three DG sections per animal. PAX6-positive and PCNA-positive cells were first counted separately, followed by quantification of double-labeled PAX6/PCNA-positive cells. For Calb1 staining, Calb1-positive cells were quantified in the DG with three sections per animal. For NeuN staining, NeuN-positive cells were likewise counted in the DG with three sections per animal. For cCasp3 staining, cCasp3-positive cells were assessed in the DG (four sections per animal), the frontal cortex (FC; six non-overlapping fields per animal), and the retrosplenial cortex (RSC; six non-overlapping fields per animal).

For quantification of PAX6+, Calb1+, NeuN+, cCasp3+, and PCNA+ cells, predefined regions of interest were analyzed in each section, and cells were counted manually using Adobe Photoshop 22.0.0 (Adobe Systems Software Ireland Limited, Dublin, Ireland), with only minimal adjustment of image contrast. For each animal, the mean cell count across all analyzed sections was calculated. These individual animal means were then normalized to the mean value of the normoxic control group (set to 100%), and group data were expressed as the mean of each experimental group relative to this control reference.

#### 2.7.2. Apoptosis Imaging and Evaluation

TUNEL-positive cells were evaluated in the frontal cortex (FC), retrosplenial cortex (RSC), hypothalamus (HTh), thalamus (Th), and hippocampus. For each animal, six non-overlapping fields were selected and analyzed in FC and RSC, and four non-overlapping fields per animal were evaluated in thalamus, and hypothalamus. The dentate gyrus (DG), including the hilum, was evaluated for a total of four DGs. For quantification of apoptotic cells, TUNEL-labeled sections counterstained with Methyl Green were analyzed using ImageJ (version 1.53). Digital images were processed with the Color Deconvolution plugin (using the H DAB) to separate Methyl Green-stained nuclei from TUNEL-positive signals. After background subtraction and automated thresholding with the Otsu method, a watershed algorithm was applied to the nuclear masks to resolve overlapping cells prior to quantification. For each animal, the mean cell count across all analyzed sections was calculated. These individual animal means were then normalized to the mean value of the normoxic control group (set to 100%), and group data were expressed as the mean of each experimental group relative to this control reference.

### 2.8. Statistical Analyses

Before study initiation and ethics approval, the required sample size per experimental group was determined a priori using G*Power software (version 3.1.2), and the resulting group sizes were adhered to throughout the study. For graphical presentation and statistical evaluation, data were displayed as box-and-whisker plots, in which the box represents the interquartile range and the horizontal line within the box denotes the median, while the whiskers illustrate the data range beyond the first and third quartiles.

Group differences were initially assessed using one-way analysis of variance (ANOVA). When the assumption of normality was not met, the non-parametric Kruskal–Wallis test was applied instead. Subsequent multiple pairwise comparisons were chosen according to the outcome of the omnibus test and performed using Bonferroni or Dunn’s post hoc procedures as appropriate. A *p*-value below 0.05 was considered statistically significant. All statistical analyses and figure preparations were conducted with GraphPad Prism (version 8.0; GraphPad Software, La Jolla, CA, USA).

## 3. Results

### 3.1. Modulation of Oxidative Stress Markers by Dexmedetomidine

**Figure 1:**
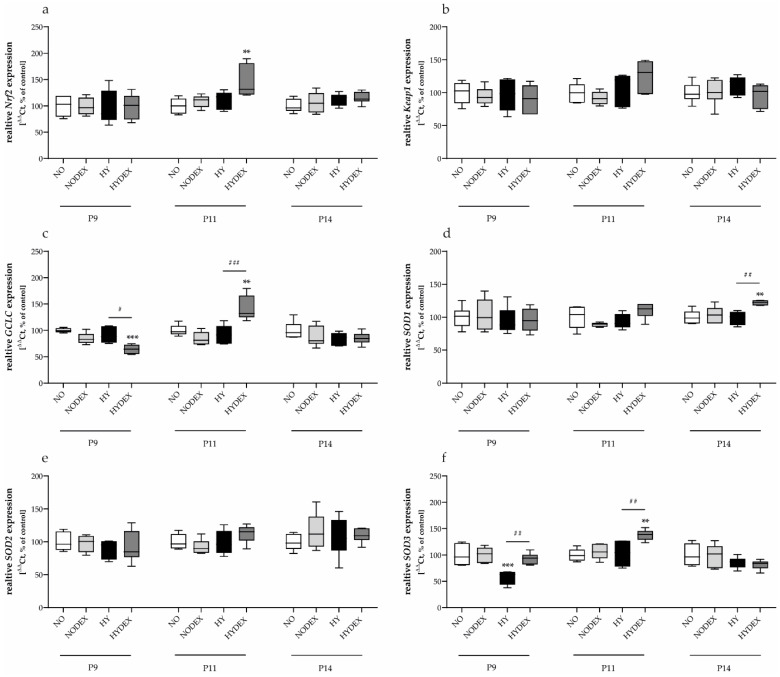
Quantification of oxidative stress regulating transcripts for (**a**) *Nrf2*, (**b**) *Keap1*, (**c**) *GCLC*, (**d**) *SOD1*, (**e**) *SOD2*, and (**f**) *SOD3* of whole cerebrum after 24 h oxygen exposure (P6 to P7) and recovery until for P9, P11, and P14 using qPCR. Data are normalized to the level of mouse pups exposed to normoxia at each time point (control 100%, NO, 21% O_2_, white bars) with verum groups hyperoxia (HY, 80% O_2_, black), normoxia with DEX 5 µg/kg (NODEX, light grey), and hyperoxia with DEX 5 µg/kg (HYDEX, dark grey). Data are presented as Box-Whisker-Plots with *n* = 6 per group. ** *p* < 0.01, *** *p* < 0.001 vs. normoxia; ^#^ *p* < 0.05, ^##^ *p* < 0.01, ^###^ *p* < 0.001 vs. hyperoxia (ANOVA; Kruskal–Wallis).

To evaluate the impact of hyperoxia and the potential protective role of dexmedetomidine (DEX) on the antioxidant defense system, we analyzed the expression of key oxidative stress markers across three postnatal time points (Figure 1). While the transcript levels of *Nrf2*, *Keap1*, *GCLC*, *SOD1*, and *SOD2* (Figure 1a–e) remained largely stable across the observation period, exposure to hyperoxia (HY) triggered a restricted immediate response defined by the significant downregulation of *SOD3* at P9 (Figure 1f).

In contrast, DEX administration under hyperoxic conditions (HYDEX) elicited a robust antioxidant signature. While *GCLC* transcription was initially suppressed at P9, it was significantly upregulated by P11 (Figure 1c). This mid-term response was accompanied by a marked increase in *Nrf2* expression at P11 (Figure 1a). Furthermore, DEX treatment led to a sustained elevation of *SOD3* levels at both P9 and P11 (Figure 1f), followed by a late-stage induction of *SOD1* at P14 (Figure 1d). No significant alterations were observed for *Keap1* or *SOD2* in the HYDEX group (Figure 1b,e).

### 3.2. Attenuation of Hyperoxia-Induced Apoptosis and Autophagic Dysregulation

**Figure 2:**
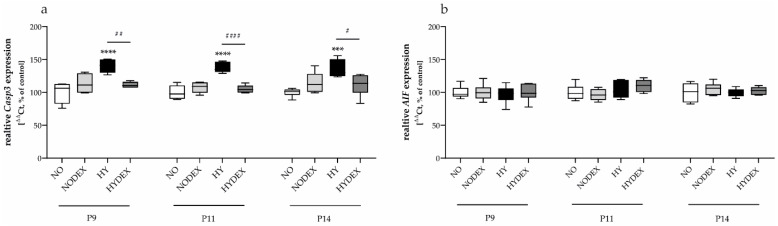
Quantification of apoptosis regulating transcripts for (**a**) *Casp3* and (**b**) *AIF* of whole cerebrum after 24 h oxygen exposure (P6 to P7) and recovery until for P9, P11, and P14 using qPCR. Data are normalized to the level of mouse pups exposed to normoxia at each time point (control 100%, NO, 21% O_2_, white bars) with verum groups hyperoxia (HY, 80% O_2_, black), normoxia with DEX 5 µg/kg (NODEX, light grey), and hyperoxia with DEX 5 µg/kg (HYDEX, dark grey). Data are presented as Box-Whisker-Plots with *n* = 6 per group. *** *p* < 0.001, **** *p* < 0.0001 vs. normoxia; ^#^ *p* < 0.05, ^##^ *p* < 0.01, ^####^ *p* < 0.0001 vs. hyperoxia (ANOVA; Kruskal–Wallis).

**Figure 3:**
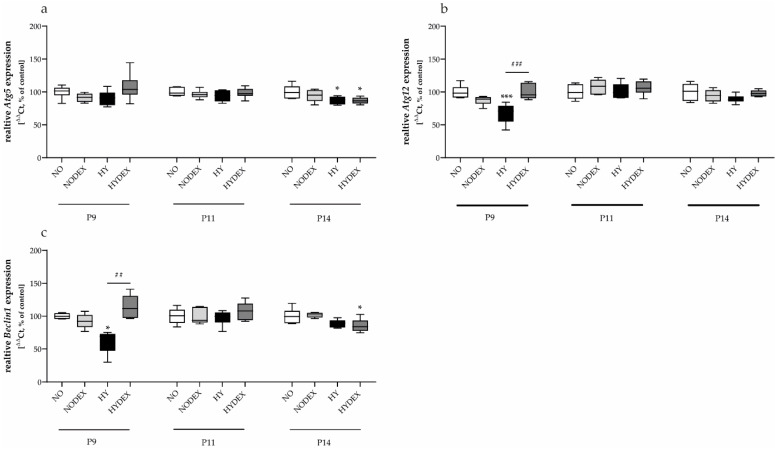
Quantification of autophagy regulating transcripts for (**a**) *Atg5*, (**b**) *Atg12*, and (**c**) *Beclin1* of whole cerebrum after 24 h oxygen exposure (P6 to P7) and recovery until for P9, P11, and P14 using qPCR. Data are normalized to the level of mouse pups exposed to normoxia at each time point (control 100%, NO, 21% O_2_, white bars) with verum groups hyperoxia (HY, 80% O_2_, black), normoxia with DEX 5 µg/kg (NODEX, light grey), and hyperoxia with DEX 5 µg/kg (HYDEX, dark grey). Data are presented as Box-Whisker-Plots with *n* = 6 per group. * *p* < 0.05, *** *p* < 0.001 vs. normoxia; ^##^ *p* < 0.01, ^###^ *p* < 0.001 vs. hyperoxia (ANOVA; Kruskal–Wallis).

High oxygen tension significantly triggered programmed cell death pathways; a phenomenon effectively mitigated by DEX (Figure 2 and Figure 3).

#### 3.2.1. Apoptosis

Hyperoxia exposure induced a persistent pro-apoptotic state, evidenced by the significant upregulation of *Casp3* mRNA from P9 through P14 (Figure 2a). This was substantiated by immunohistochemical analyses showing a surge in TUNEL-positive cells within the frontal cortex (FC) and retrosplenial cortex (RSC) at all time points, and the hypothalamus (HTh) until P11 (Table 1). Similarly, cleaved caspase-3 (cCasp3) expression was markedly elevated in the FC, RSC, and hippocampus (HC) throughout the study (Table 2). Notably, *AIF* expression remained stable under HY conditions (Figure 2b).

Treatment with DEX (HYDEX) significantly reversed these trends. A consistent reduction in *Casp3* transcripts was observed across all measurement time points. This molecular rescue was mirrored at the cellular level, with a decrease in TUNEL-positive cells (Table 1) in the FC and RSC (P9–P14) and the HC (P9). Furthermore, DEX treatment effectively reduced cCasp3-positive cell counts (Table 2) in all analyzed regions (FC, RSC, and HC) from P9 to P14, indicating a widespread anti-apoptotic effect.

#### 3.2.2. Autophagy

The autophagic machinery, also, was sensitive to oxygen fluctuations (Figure 3). Hyperoxia led to a transient downregulation of *Atg12* (Figure 3b) and Beclin1 at P9 (Figure 3c), followed by a late-stage reduction in *Atg5* at P14 (Figure 3a). DEX administration (HYDEX) appeared to prime the autophagic response, significantly increasing *Atg12* and *Beclin1* expression at P9 (Figure 3b,c), although *Atg5* levels remained low at P14, similar to the HY group (Figure 3a).

### 3.3. Preservation of the Radial Glia Pool and Glial Identity

**Figure 4:**
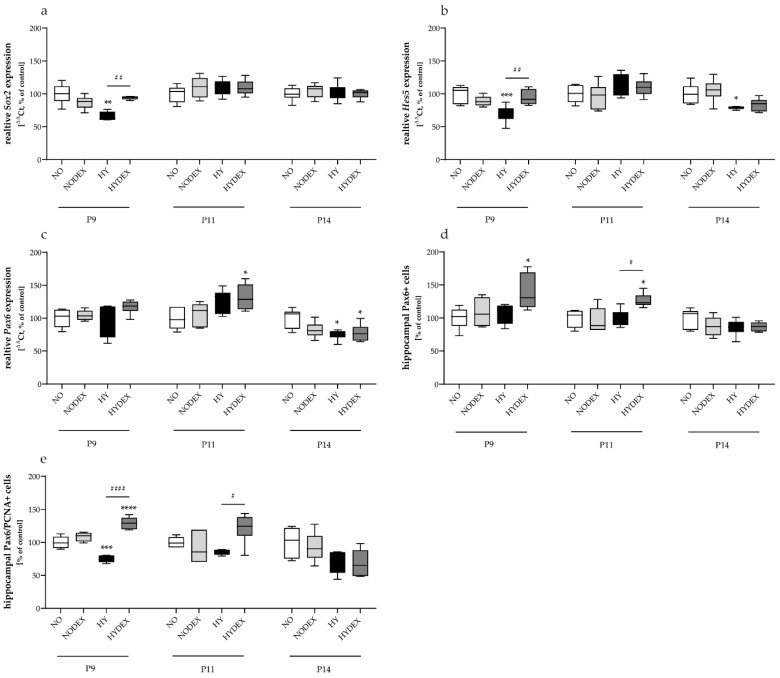
Quantification of radial glia pool regulating transcripts for (**a**) *Sox2*, (**b**) *Hes5*, and (**c**) *Pax6* of whole cerebrum and quantification of (**d**) Pax6 and (**e**) Pax6/PCNA immunostaining of dentate gyrus after 24 h oxygen exposure (P6 to P7) and recovery until for P9, P11, and P14 using qPCR. Data are normalized to the level of mouse pups exposed to normoxia at each time point (control 100%, NO, 21% O_2_, white bars) with verum groups hyperoxia (HY, 80% O_2_, black), normoxia with DEX 5 µg/kg (NODEX, light grey), and hyperoxia with DEX 5 µg/kg (HYDEX, dark grey). Data are presented as Box-Whisker-Plots with *n* = 6 per group. * *p* < 0.05, ** *p* < 0.01, *** *p* < 0.001, **** *p* < 0.0001 vs. normoxia; ^#^ *p* < 0.05, ^##^ *p* < 0.01, ^####^ *p* < 0.0001 vs. hyperoxia (ANOVA; Kruskal–Wallis).

**Figure 5:**
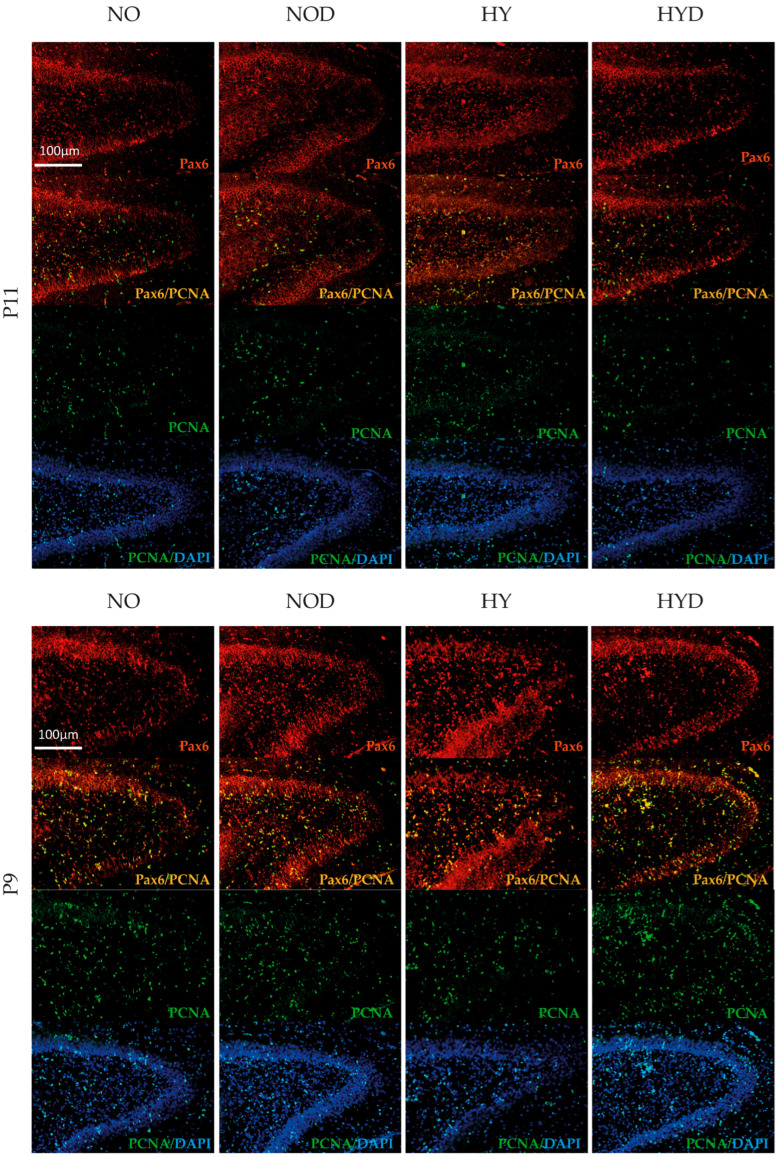

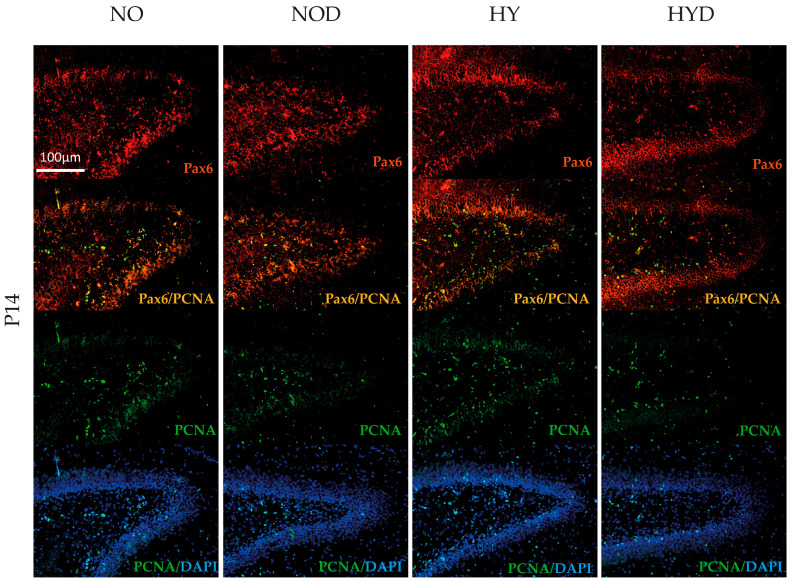
Representative sections of the neonatal hippocampus with the dentate gyrus at postnatal day 9 (P9), P11, and P14, after a recovery phase following a 24 h hyperoxia (HY) exposure period (P6–P7) labeled with Pax6 (red), double labeled Pax6/PCNA (orange), PCNA (green) and DAPI (blue), are shown with normoxia (NO, 21% O_2_), with verum groups hyperoxia (HY, 80% O_2_), normoxia with DEX 5 µg/kg (NOD), and hyperoxia with DEX 5 µg/kg (HYD).

**Figure 6:**
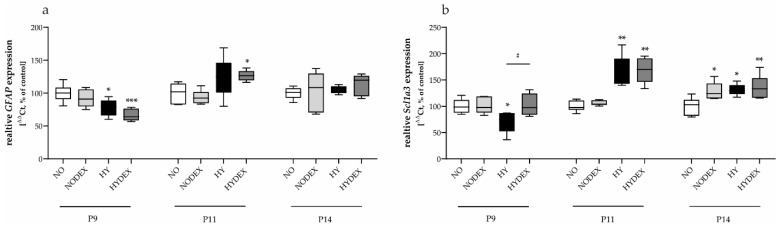
Quantification of glia pool regulating transcripts for (**a**) *GFAP* and (**b**) *Scl1a3*, of whole cerebrum after 24 h oxygen exposure (P6 to P7) and recovery until for P9, P11, and P14 using qPCR. Data are normalized to the level of mouse pups exposed to normoxia at each time point (control 100%, NO, 21% O_2_, white bars) with verum groups hyperoxia (HY, 80% O_2_, black), normoxia with DEX 5 µg/kg (NODEX, light grey), and hyperoxia with DEX 5 µg/kg (HYDEX, dark grey). Data are presented as Box-Whisker-Plots with *n* = 6 per group. * *p* < 0.05, ** *p* < 0.01, *** *p* < 0.001 vs. normoxia; ^#^ *p* < 0.05 vs. hyperoxia (ANOVA; Kruskal–Wallis).

The impact of hyperoxia on early neural progenitor cells and subsequent glial development was profound, with DEX showing significant regenerative potential (Figure 4, Figure 5 and Figure 6).

Hyperoxia significantly impaired the radial glia-like cell population, as shown by the downregulation of *Sox2* and *Hes5* at P9 (Figure 4a,b) and *Pax6* at P14 (Figure 4c). Immunohistochemical evaluation of the dentate gyrus (DG) confirmed reduction in proliferating Pax6/PCNA-double-positive progenitors at P9 (Figure 4e), while single Pax6 labeled cells were not reduced (Figure 4d).

Representative immunohistochemical analysis corroborated the quantitative findings within the progenitor niche of the dentate gyrus (DG). At P9, while the overall intensity of the Pax6-labeled pool appeared stable, hyperoxia (HY) was associated with a selective reduction in actively dividing progenitors, as indicated by a significant decrease in Pax6/PCNA double-positive cells (Figure 5, upper panel). In the subsequent developmental stages, hyperoxia exposure showed no discernible influence on the Pax6+ lineage; both the total number of Pax6+ cells and their proliferating (Pax6/PCNA+) fractions remained comparable to control levels at P11 (Figure 5, middle panel) and P14 (Figure 5, lower panel).

Furthermore, glial markers *GFAP* and *Slc1a3* were downregulated at P9, though *Slc1a3* showed a compensatory increase by P11–P14 (Figure 6a,b).

DEX treatment (HYDEX) successfully counteracted these deficits. An immediate upregulation of *Sox2* and *Hes5* was observed at P9 (Figure 4a,b). In the DG, DEX promoted the expansion of the progenitor pool, evidenced by an increase in Pax6-positive cells at P11 (Figure 4d) and a significant rise in Pax6/PCNA-positive proliferating cells at both P9 and P11 (Figure 4e).

DEX administration was associated with changes in the progenitor population under hyperoxic conditions. At P9, DEX treatment neutralized the hyperoxia-induced inhibitory effect, acting as a potent stimulus that substantially elevated the density of mitotic Pax6-positive cells (Figure 5, upper panel). This stimulatory influence persisted until P11 (Figure 5, middle panel); DEX significantly increased the counts of both the total Pax6+ population and the proliferating Pax6/PCNA+ progenitors. By P14 (Figure 5, lower panel), these lineage-specific effects reached a plateau, with no further significant differences observed in the mitotic or non-mitotic Pax6+ populations compared to the HY group.

Additionally, DEX restored *GFAP* levels to baseline (Figure 6a) and accelerated the normalization of *Slc1a3* expression by P9 (Figure 6b).

### 3.4. Impact on Interneuron Lineages, Neurotrophins, and Neuronal Plasticity

**Figure 7:**
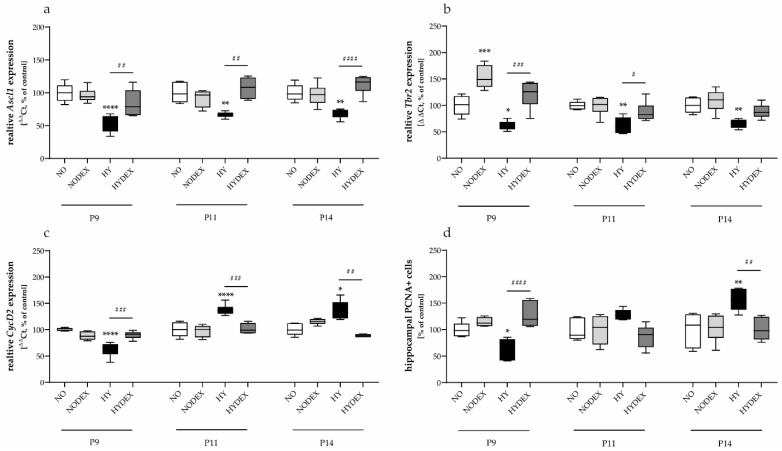
Quantification of mitotic immature neuron regulating transcripts for (**a**) *Ascl1*, (**b**) *Tbr2*, (**c**) *CycD2*, and of whole cerebrum and quantification of (**d**) PCNA immunostaining of dentate gyrus after 24 h oxygen exposure (P6 to P7) and recovery until for P9, P11, and P14 using qPCR. Data are normalized to the level of mouse pups exposed to normoxia at each time point (control 100%, NO, 21% O_2_, white bars) with verum groups hyperoxia (HY, 80% O_2_, black), normoxia with DEX 5 µg/kg (NODEX, light grey), and hyperoxia with DEX 5 µg/kg (HYDEX, dark grey). Data are presented as Box-Whisker-Plots with *n* = 6 per group. * *p* < 0.05, ** *p* < 0.01, *** *p* < 0.001, **** *p* < 0.0001 vs. normoxia; ^#^ *p* < 0.05, ^##^ *p* < 0.01, ^###^ *p* < 0.001, ^####^ *p* < 0.0001 vs. hyperoxia (ANOVA; Kruskal–Wallis).

**Figure 8:**
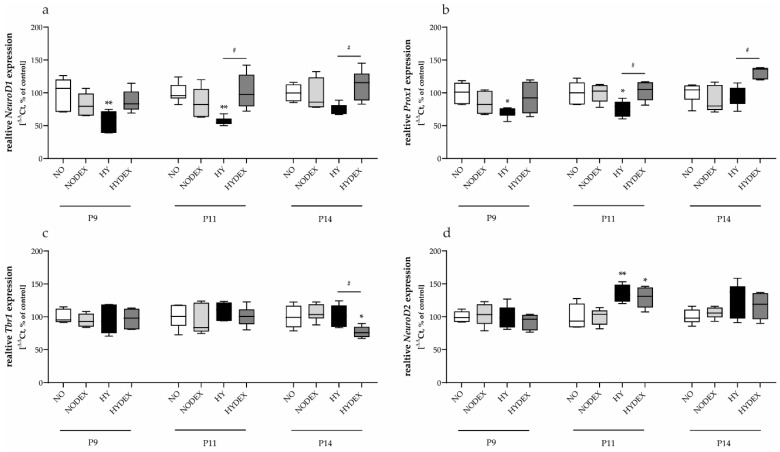
Quantification of post-mitotic immature neuron regulating transcripts for (**a**) *NeuroD1*, (**b**) *Prox1*, (**c**) *Tbr1*, and (**d**) *NeuroD2* of whole cerebrum and quantification of d) PCNA immunostaining of dentate gyrus after 24 h oxygen exposure (P6 to P7) and recovery until for P9, P11, and P14 using qPCR. Data are normalized to the level of mouse pups exposed to normoxia at each time point (control 100%, NO, 21% O_2_, white bars) with verum groups hyperoxia (HY, 80% O_2_, black), normoxia with DEX 5 µg/kg (NODEX, light grey), and hyperoxia with DEX 5 µg/kg (HYDEX, dark grey). Data are presented as Box-Whisker-Plots with *n* = 6 per group. * *p* < 0.05, ** *p* < 0.01 vs. normoxia; ^#^ *p* < 0.05 vs. hyperoxia (ANOVA; Kruskal–Wallis).

**Figure 9:**
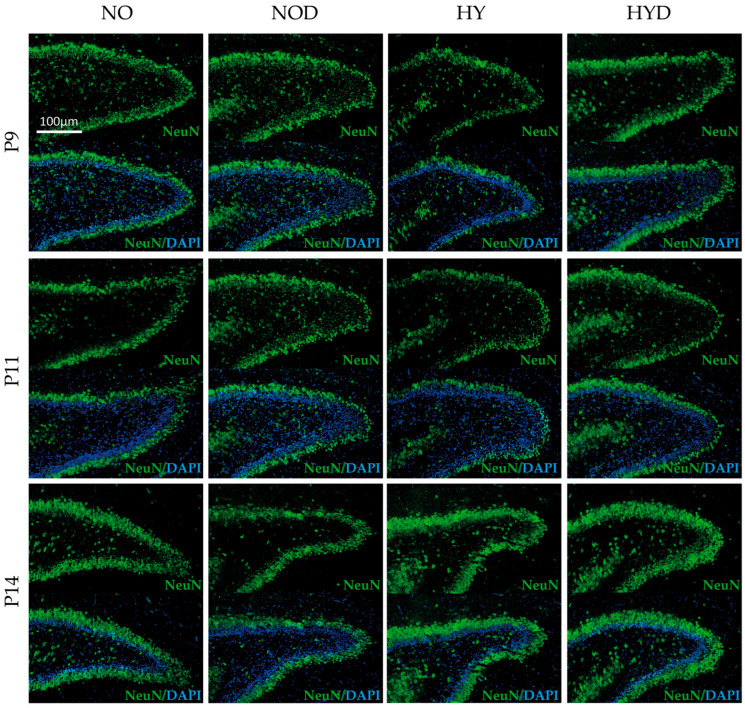
Representative sections of the neonatal hippocampus with the dentate gyrus at postnatal day 9 (P9), P11, and P14, after a recovery phase following a 24 h hyperoxia (HY) exposure period (P6–P7) labeled with NeuN (green) and DAPI (blue), are shown with normoxia (NO, 21% O_2_), with verum groups hyperoxia (HY, 80% O_2_), normoxia with DEX 5 µg/kg (NOD), and hyperoxia with DEX 5 µg/kg (HYD).

**Figure 10:**
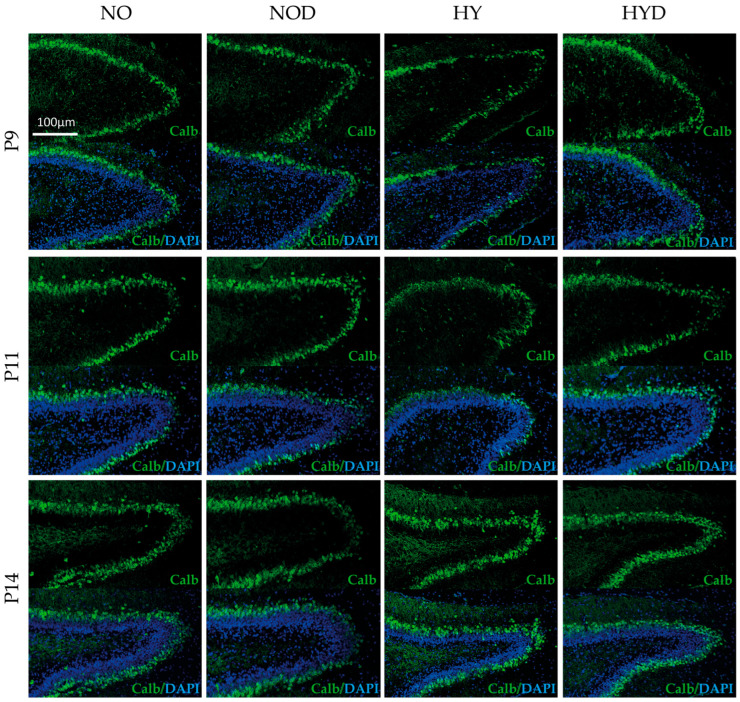
Representative sections of the neonatal hippocampus with the dentate gyrus at postnatal day 9 (P9), P11, and P14, after a recovery phase following a 24 h hyperoxia (HY) exposure period (P6–P7) labeled with Calb1 (green) and DAPI (blue), are shown with normoxia (NO, 21% O_2_), with verum groups hyperoxia (HY, 80% O_2_), normoxia with DEX 5 µg/kg (NOD), and hyperoxia with DEX 5 µg/kg (HYD).

We further examined the progression of interneuron development and the supportive neurotrophic environment required for neuronal maturation (Figure 7, Figure 8, Figure 9 and Figure 10).

#### 3.4.1. Mitotic and Postmitotic Interneurons

Hyperoxia strongly inhibited interneuron neurogenesis, suppressing mitotic markers *Ascl1* and *Tbr2* from P9 to P14 (Figure 7a,b) and reducing PCNA-positive cells in the DG at P9 (Figure 7d). Postmitotic markers *NeuroD1* and *Prox1* were likewise downregulated at P9 and P11 (Figure 8a,b). DEX treatment (HYDEX) effectively reversed these trends, stimulating a robust increase in *Ascl1* (P9–P14, Figure 7a), *Tbr2* (P9–P11, Figure 7b), and both *NeuroD1* and *Prox1* (P11–P14, Figure 8a,b).

Hyperoxia severely diminished interneuron neurogenesis, as indicated by suppression of mitotic markers *Ascl1* and *Tbr2* from P9 to P14 (Figure 7a,b). This early suppression was further reflected in the expression of the cell cycle regulator *CycD2*, which was significantly downregulated at P9, followed by a late-stage compensatory increase at P11 and P14 compared to normoxic controls (Figure 7c). Similarly, PCNA expression in the hippocampus (HC) showed a significant reduction at P9 before reaching higher levels than controls at P14, with no significant changes observed at P11 (Figure 7d). Assessment of general neuronal proliferation via PCNA immunohistochemistry revealed a distinct temporal response to oxygen stress. At P9, hyperoxia exposure (HY) led to a broad suppression of the total PCNA-positive proliferative population within the dentate gyrus (Figure 5, upper panel). While this proliferative activity seemed unaffected and remained within the range of control levels at P11 (Figure 5, middle panel), the P14 stage was characterized by a significant reactive surge in cellular turnover (Figure 5, lower panel). Specifically, hyperoxia-exposed subjects exhibited an increase in total PCNA-positive cell counts that exceeded baseline levels, suggesting a delayed or compensatory proliferative response to the preceding neonatal injury.

Postmitotic markers *NeuroD1* and *Prox1*, likewise, were downregulated at P9 and P11 (Figure 8a,b). Interestingly, while the postmitotic marker *Tbr1* remained unchanged by hyperoxia across all time points (P9–P14, Figure 8c), *NeuroD2* expression showed a transient but significant upregulation at P11 in hyperoxic animals (Figure 8d).

DEX treatment (HYDEX) effectively modulated these trends. In the mitotic compartment, DEX stimulated a robust early increase in *Ascl1* (P9–P14, Figure 7a), *Tbr2* (P9–P11, Figure 7b), and both *CycD2* and PCNA (HC) at P9, thereby reversing the initial hyperoxia-induced suppression (Figure 7c,d). However, this early stimulation was followed by a subsequent decline in *CycD2* and PCNA (HC) levels at P14. DEX administration significantly modulated the proliferative landscape under hyperoxic conditions. At P9, DEX (HYDEX) acted as a potent mitogenic stimulus, not only effectively reversing the hyperoxia-induced deficit but also significantly enhancing overall proliferative activity beyond baseline levels (Figure 5, upper panel). This early intervention appeared to stabilize cellular turnover; while hyperoxia-treated animals showed a late-stage reactive surge at P14 (Figure 5, lower panel), DEX treatment prevented this overshooting response, maintaining proliferative counts closer to physiological levels, thereby facilitating a more balanced developmental trajectory.

Regarding postmitotic markers, DEX treatment not only increased *NeuroD1* and *Prox1* (P11–P14, Figure 8a,b) but also led to a specific downregulation of *Tbr1* at P14. In contrast, *NeuroD2* levels remained largely stable and showed no significant alterations following DEX treatment compared to the hyperoxia group (Figure 8c,d).

#### 3.4.2. Mature Neuronal Markers

**Figure 11:**
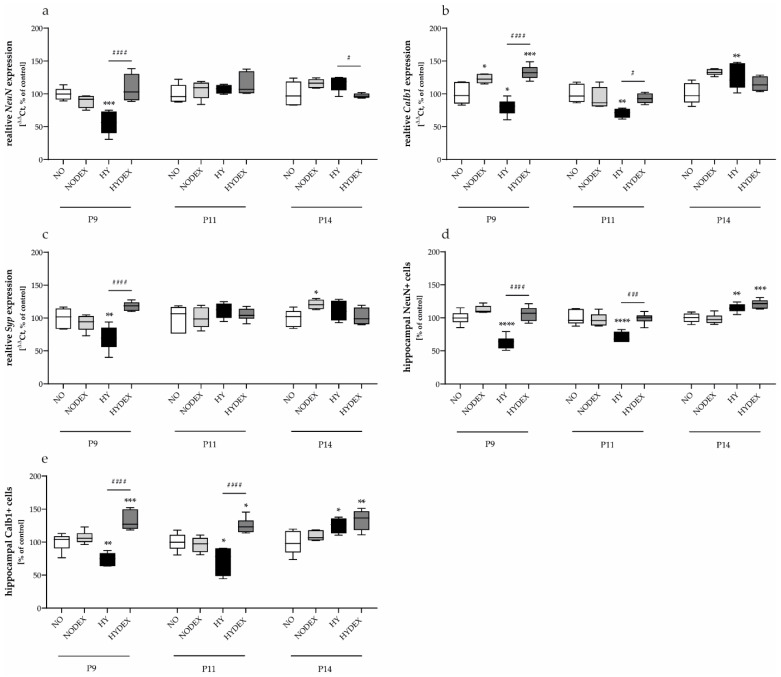
Quantification of mature neuron regulating transcripts for (**a**) *NeuN*, (**b**) *Calb1*, (**c**) *Syp*, and of whole cerebrum and quantification of (**d**) NeuN, and (**e**) Calb1 immunostaining of dentate gyrus after 24 h oxygen exposure (P6 to P7) and recovery until for P9, P11, and P14 using qPCR. Data are normalized to the level of mouse pups exposed to normoxia at each time point (control 100%, NO, 21% O_2_, white bars) with verum groups hyperoxia (HY, 80% O_2_, black), normoxia with DEX 5 µg/kg (NODEX, light grey), and hyperoxia with DEX 5 µg/kg (HYDEX, dark grey). Data are presented as Box-Whisker-Plots with *n* = 6 per group. * *p* < 0.05, ** *p* < 0.01, *** *p* < 0.001, **** *p* < 0.0001 vs. normoxia; ^#^ *p* < 0.05, ^###^ *p* < 0.001, ^####^ *p* < 0.0001 vs. hyperoxia (ANOVA; Kruskal–Wallis).

Finally, the transition to a mature neuronal phenotype was delayed by hyperoxia, as indicated by decreased NeuN−(Figure 9) and Calb1−(Figure 10) labeled neurons in the DG and at the mRNA level at P9–P11 (Figure 11a,b). Immunohistochemical staining for the mature neuronal markers NeuN (Figure 9) and Calbindin-1 (Calb1; Figure 10) within the dentate gyrus (DG) revealed a significant disruption of the neuronal maturation cascade under hyperoxic conditions. At postnatal day 9 (P9), the hyperoxia (HY) group exhibited a marked rarefaction of both NeuN-positive (Figure 9, upper panel) and Calb1-positive cell populations within the granular cell layer. This deficit in neuronal density persisted through P11 (Figure 9, middle panel), suggesting a sustained suppression of terminal neuronal differentiation. By P14 (Figure 9, lower panel), however, representative images indicated a subjective restoration to control-like levels, reflecting a delayed onset of spontaneous normalization in the expression of mature neuronal markers despite the preceding hyperoxic insult. Interestingly, a late-stage mRNA increase in these markers was observed at P14 in the HY group (Figure 11b–d). *Syp* mRNA transcription was reduced by hyperoxia at P9 (Figure 11e).

DEX treatment (HYDEX) accelerated the maturation process, resulting in a significant increase in NeuN and Calbindin-1 (Calb1) protein expression in the DG at P9 and P11 (Figure 11c,d), as well as a reversal of the hyperoxia-induced reduction of the synaptic marker *Syp* at P9 (Figure 11e). DEX administration effectively counteracted the hyperoxia-induced impairment of neuronal maturation. Representative sections demonstrated an impressive preservation of neuronal differentiation, characterized by a substantially higher density of NeuN− (Figure 9) and Calb1-positive cells (Figure 10) compared to the oxygen-exposed vehicle group. This protective effect of DEX was consistently observed across all analyzed time points (P9, P11, and P14).

#### 3.4.3. Neurotrophic Support and Plasticity

**Figure 12:**
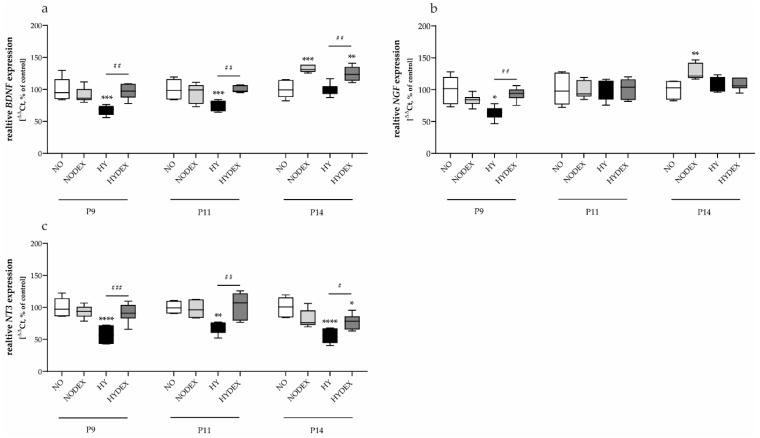
Quantification of neurotrophin transcripts for (**a**) *BDNF*, (**b**) *NGF*, and (**c**) *NT3* of whole cerebrum after 24 h oxygen exposure (P6 to P7) and recovery until for P9, P11, and P14 using qPCR. Data are normalized to the level of mouse pups exposed to normoxia at each time point (control 100%, NO, 21% O_2_, white bars) with verum groups hyperoxia (HY, 80% O_2_, black), normoxia with DEX 5 µg/kg (NODEX, light grey), and hyperoxia with DEX 5 µg/kg (HYDEX, dark grey). Data are presented as Box-Whisker-Plots with *n* = 6 per group. * *p* < 0.05, ** *p* < 0.01, *** *p* < 0.001, **** *p* < 0.0001 vs. normoxia; ^#^ *p* < 0.05, ^##^ *p* < 0.01, ^###^ *p* < 0.001 vs. hyperoxia (ANOVA; Kruskal–Wallis).

Neurotrophic signaling, essential for survival and differentiation, was significantly hampered by HY, with *BDNF*, *NGF*, and *NT3* showing marked downregulation at multiple time points (Figure 12a–c). DEX administration (HYDEX) significantly boosted the expression of all three neurotrophins (*BDNF*, *NGF*, and *NT3*) throughout the entire observation period (Figure 12a–c).

**Figure 13:**
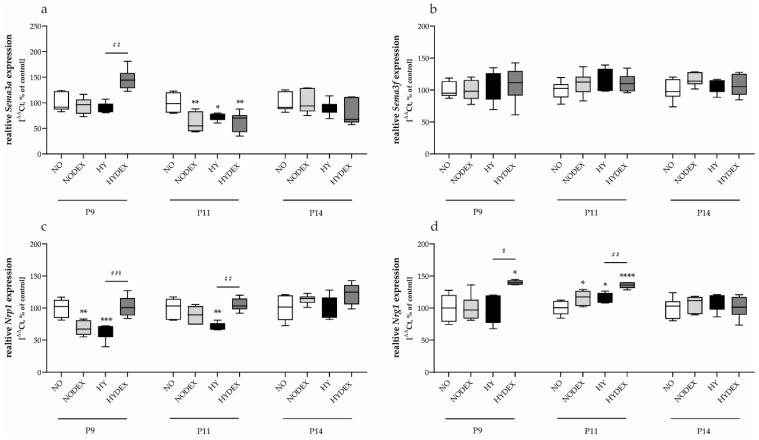
Quantification of neuronal plasticity regulating transcripts for (**a**) *Sema3a*, (**b**) *Sema3f*, (**c**) *Nrp1*, and (**d**) *Nrg1* of whole cerebrum after 24 h oxygen exposure (P6 to P7) and recovery until for P9, P11, and P14 using qPCR. Data are normalized to the level of mouse pups exposed to normoxia at each time point (control 100%, NO, 21% O_2_, white bars) with verum groups hyperoxia (HY, 80% O_2_, black), normoxia with DEX 5 µg/kg (NODEX, light grey), and hyperoxia with DEX 5 µg/kg (HYDEX, dark grey). Data are presented as Box-Whisker-Plots with *n* = 6 per group. * *p* < 0.05, ** *p* < 0.01, *** *p* < 0.001, **** *p* < 0.0001 vs. normoxia; ^#^ *p* < 0.05, ^##^ *p* < 0.01, ^###^ *p* < 0.001 vs. hyperoxia (ANOVA; Kruskal–Wallis).

This neurotrophic environment likely supported the observed changes in neuronal plasticity markers (Figure 13). While hyperoxia suppressed *Nrp1* (P9 and P11) and *Sema3a* (P11) (Figure 13a,c), DEX treatment (HYDEX) led to a significant upregulation of *Sema3a*, *Nrp1*, and *Nrg1* at P9 and/or P11 (Figure 13a,c,d), suggesting an enhanced capacity for circuit remodeling and repair. Remarkably, the *Sema3f* expression remained stable under both HY and DEX conditions. (Figure 13b).

### 3.5. Integrative Summary of Hyperoxia-Induced Impairment and Dexmedetomidine Neuroprotection

**Figure 14:**
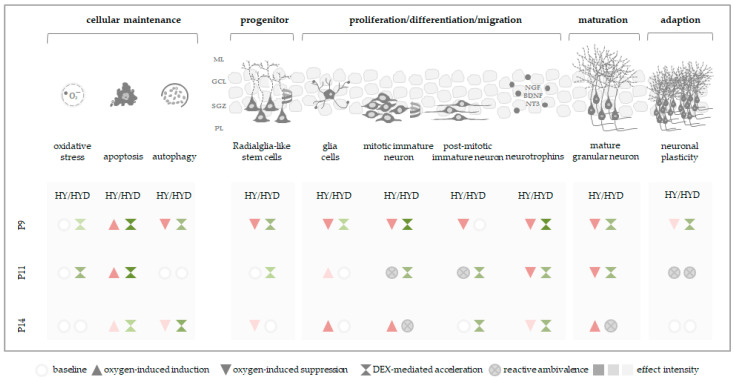
Graphical summary of the spatiotemporal effects of hyperoxia (HY) and dexmedetomidine (HYD) on the developing rat brain. The overview illustrates the shift from oxygen-induced suppression/induction toward DEX-mediated acceleration of recovery and developmental normalization across cellular maintenance, progenitor maintenance, proliferation, maturation, and adaptation phases.

To provide a comprehensive overview of the complex interactions between hyperoxic injury and the protective potential of dexmedetomidine (DEX), our findings are synthesized into a holistic temporal model covering the neonatal and juvenile developmental phases (Figure 14).

While oxidative stress markers exhibited a delayed or mild response, programmed cell death (apoptosis) was acutely and persistently induced from P9 to P14. This was accompanied by a significant inhibition of autophagic flux, particularly immediately following oxygen exposure at P9. DEX administration (HYD) demonstrated robust regenerative efficacy; it not only accelerated the normalization of apoptotic signaling but also activated antioxidant defenses and autophagy, effectively shifting the cellular environment from injury toward maintenance.

Neuronal maturation and differentiation were severely compromised by hyperoxia, as evidenced by a marked decline in the radial glia-like stem cell population and the broad suppression of both mitotic and post-mitotic immature neurons at P9. Although the system exhibited “reactive ambivalence” at later stages (P11–P14), characterized by irregular induction or sustained suppression, natural recovery was insufficient to restore baseline growth patterns. DEX treatment (HYD) served as a developmental catalyst, significantly accelerating progenitor proliferation and facilitating an orderly progression through the interneuron lineage.

The supportive niche essential for neuronal maturation was significantly impaired under hyperoxia, reflected by the sustained downregulation of critical neurotrophins and a corresponding delay in the appearance of mature granule cells at P9 and P11. Furthermore, the capacity for neuronal plasticity and adaptation was markedly diminished during the early phase. DEX administration effectively bypassed these developmental blocks. By bolstering neurotrophic support and enhancing plasticity signaling, DEX promoted granule cell maturation, ensuring the preservation of structural and functional brain adaptation despite hyperoxic stress.

## 4. Discussion

The present study confirms that short-term neonatal hyperoxia does not merely induce transient oxidative stress but moreover initiates a sustained dysmaturational cascade affecting multiple hierarchical levels of brain development. While classical antioxidant transcriptional responses remained relatively modest, the pronounced and persistent activation of apoptotic pathways indicates that oxidative injury is rapidly translated into structural cellular loss. This apparent dissociation suggests that even subtle redox imbalances in the immature brain are sufficient to trigger downstream degenerative programs without inducing a robust transcriptional antioxidant response. Importantly, hyperoxia perturbed not only cell survival but also developmental timing. The early suppression of progenitor proliferation, interneuron lineage progression, and neuronal maturation, followed by partial or compensatory rebounds at later stages, reflects a pattern of reactive maladaptation. This phenomenon aligns with the concept of developmental dysmaturation, where injury alters the trajectory rather than causing uniform loss. The late increases in proliferative and maturational markers likely represent an attempt to restore cellular deficits; however, such delayed responses are unlikely to recapitulate the tightly coordinated sequence of neurodevelopmental events required for functional circuit formation.

The mechanistic basis of this observed dysmaturation lies in a biphasic injury dynamic, where the initial suppression of neural progenitor cell (NPC) proliferation and interneuron lineage progression is driven by oxidative stress-mediated pathways, particularly aberrant GSK3β signaling [17,28,46]. This phase is marked by the downregulation of key transcription factors, such as PAX6 and Tbr2, and disruption of vascular–neural coupling, compromising the neurovascular niche critical for NPC maintenance [17,23,40,46,47,48]. The subsequent failure of late proliferative rebounds reflects temporal dysregulation, as core developmental programs such as SHH and Wnt signaling are irreversibly altered, causing delayed responses to occur outside critical windows for circuit refinement [12,20,28,47]. As a result, reactive maladaptation leads to structurally abnormal networks characterized by impaired synaptogenesis, altered GABA homeostasis, and reduced dendritic complexity [21,49]. Unlike adaptive plasticity, this process represents a failure to recapitulate the neurodevelopmental sequence, establishing lasting cellular substrates for cognitive and motor deficits observed clinically [12,17,20,49,50].

### 4.1. Orchestration of Antioxidant Defenses and Autophagic Priming

A key finding is the temporal mismatch between hyperoxic insult and the endogenous antioxidant response, reflecting the intrinsic immaturity of the preterm brain, which lacks efficient levels of enzymes such as SOD, glutathione peroxidase, and catalase [3,51]. Hyperoxia induced an immediate downregulation of SOD3, shifting the redox balance toward reactive oxygen and nitrating species [46,52,53], while the broader antioxidant system, including Nrf2, Keap1, and GCLC, remained largely unresponsive. The impact of this imbalance is indicated by the delayed or insufficient activation of the Nrf2–Keap1 pathway in the neonatal brain [54,55]. In contrast, DEX elicited a proactive antioxidant response, with early SOD3 induction followed by mid-term upregulation of Nrf2 and GCLC. This pattern suggests that DEX not only scavenges ROS but actively recalibrates redox sensing, likely by promoting Nrf2 nuclear translocation, potentially via MALAT1/miR-140-5p signaling [56], thereby restoring glutathione homeostasis and reducing lipid peroxidation [39,41,42]. Additionally, hyperoxia-induced suppression of Beclin1 and Atg12 at P9 indicates impaired basal autophagy and reduced clearance of damaged organelles [57,58]. DEX counteracted this by early upregulation of these markers, enhancing proteostasis and limiting the accumulation of toxic protein aggregates and dysfunctional mitochondria, thereby attenuating downstream apoptotic signaling [57,59]. However, the later reduction of Atg5 under hyperoxia exposure suggests incomplete long-term compensation of autophagic capacity, pointing to a persistent proteostatic deficit with implications for clinical translation [26,60,61,62].

### 4.2. Mitigation of Persistent Apoptotic Signaling

Our findings show that hyperoxia-induced programmed cell death in the neonatal CNS is widespread and persists into the second postnatal week, with elevated cleaved caspase-3 and TUNEL positivity across the frontal cortex, retrosplenial cortex, and hippocampus. This regional vulnerability is consistent with established models in which 80% oxygen exposure induces neurodegeneration in key developmental structures [41,47,63]. The lack of significant AIF modulation indicates that injury primarily proceeds via caspase-dependent rather than caspase-independent mitochondrial pathways [64,65], in line with activation of Fas/CD95 and BAX-dependent cascades leading to caspase-8 and caspase-3 activation [66,67].

DEX treatment markedly reduced apoptotic markers across regions from P9 to P14, consistent with evidence that α2-adrenoceptor agonism attenuates neurodegeneration and preserves neuronal density in the developing hippocampus and cortex [23,26,41,67]. Meta-analyses further support a consistent reduction in TUNEL-positive cells across brain injury models [68]. This broad neuroprotection likely reflects combined anti-inflammatory effects and preservation of mitochondrial and blood–brain barrier integrity [39,69].

Mechanistically, the PI3K/Akt pathway appears central to DEX-mediated protection. Its activation decouples oxidative stress from apoptotic execution, as shown by increased phosphorylated Akt and Bad, restoration of the Bcl-xL/Bad ratio, and suppression of caspase-3 cleavage [70,71,72]. This α2-adrenoceptor–mediated signaling also reduces ROS and downstream inflammatory cytokines [70,73,74,75]. Emerging evidence further suggests modulation of pyroptosis and calcium homeostasis, indicating neuroprotective effects beyond the PI3K/Akt axis [73,76].

### 4.3. Preservation of the Progenitor Niche and Prevention of Reactive Ambivalence

A central finding is the dynamic vulnerability of the neural progenitor pool in the dentate gyrus. Hyperoxia induced a pronounced maturation arrest, reflected by reduced Sox2, essential for radial glia stemness and proliferation [77], and Hes5, a Notch-dependent regulator maintaining undifferentiated states [78]. This early depletion, supported by reduced mitotic Pax6/PCNA+ cells, aligns with and extends prior evidence of impaired hippocampal neurogenesis following oxygen exposure [25,40,48,79]. Mechanistically, the loss of Hes5+ cells indicates disruption of Notch signaling, promoting premature differentiation and progenitor exhaustion [80,81,82]. Translational data further suggest that such early deficits in self-renewal result in persistent impairments in neurogenesis and cognition [17].

At later stages, a pattern of reactive ambivalence emerged: initial progenitor suppression was followed by a delayed, disorganized increase in PCNA+ cells at P14. This unsynchronized proliferative rebound is consistent with ineffective neurogenesis, as increased proliferation does not translate into structural growth, indicating maladaptive circuit formation [83]. Such temporal dysregulation is known to impair hippocampal circuit organization and excitatory/inhibitory balance [28].

DEX treatment preserved progenitor dynamics by maintaining Pax6+ and Sox2+ populations at P9 and P11, thereby preventing early reservoir depletion. This effect is supported by increased Sox2 and PCNA+ expression following DEX treatment [40,84], and by evidence that DEX modulates Pax6+ progenitors to restore physiological growth trajectories after hyperoxic injury [26]. Importantly, DEX prevented the late-stage proliferative overshoot at P14, stabilizing developmental timing and cellular turnover. This normalization likely involves integrated mechanisms, including GDNF/NCAM/CREB signaling [85], inhibition of JNK and p38 MAPK pathways [86], and suppression of microglial activation and inflammatory cytokines [87].

Clinically, the ability of DEX to normalize progenitor dynamics is highly relevant for preterm infants exposed to oxygen therapy, where hyperoxia contributes to adverse neurodevelopmental outcomes, particularly within the lung–brain axis in bronchopulmonary dysplasia [14,16]. Consistent with this, DEX exhibits a multifaceted neuroprotective profile, reducing oxidative stress, limiting lipid peroxidation, and preserving transcriptional programs critical for neuronal development [39,41,88].

### 4.4. Restoration of the Neurotrophic Niche and Neuronal Plasticity

This study demonstrates that DEX confers robust, multimodal neuroprotection against hyperoxia-induced hippocampal injury, primarily by restoring neurotrophic signaling and promoting neuronal maturation and plasticity. Hyperoxia markedly delayed the transition from immature progenitors to mature neurons (NeuN+/Calb1+), coinciding with sustained downregulation of BDNF, NGF, and NT3. This neurotrophic deficit impaired synaptogenesis and dendritic development, reflected by reduced Synaptophysin (Syp) levels [17,88], consistent with reports of decreased PSD95 and GAP43 and subsequent long-term cognitive deficits in neonatal models [21,28,40,47,89].

DEX restored neurotrophin expression throughout P9–P14, facilitating granule cell maturation. Mechanistically, DEX enhances astrocytic BDNF via ERK-dependent signaling and activates the BDNF/TrkB pathway, reducing apoptosis and improving functional outcomes [90,91]. Sustained neurotrophic support also preserved key developmental markers (e.g., PSA-NCAM+, Nestin+, Sox2, Tbr1/2, Prox1) suppressed by hyperoxia [26,40].

In parallel, DEX modulated plasticity-related pathways. Hyperoxia reduced expression of Sema3a, Nrp1, and Nrg1, whereas DEX restored their levels [40]. The Sema3a/neuropilin-1 axis is critical for activity-dependent synaptic plasticity and AMPA receptor trafficking [92]. DEX further supports hippocampal neurogenesis and spatial learning via GDNF/NCAM/CREB signaling and NMDA receptor phosphorylation, while preserving Purkinje cells and cerebellar progenitors [26,85,93].

In terms of translational relevance, DEX seems to have remarkable pharmacological properties not only for efficacy for neuroprotection in injury-stimulated animals, but also for safety in control animals. It is of high relevance for the goal of defining a neuroprotective compound for the clinical use in a vulnerable patient population represented in newborn and preterm infants, that it will not itself cause damage in cells, brains, and patients [38]. Notably, in our experiments, DEX appeared to be harmless in all experimental readouts of cellular development, survival, and anti-oxidant functions in the brains of newborn rats. In comparison to many other CNS protective agents, this beneficial efficacy-harm-profile of DEX is very promising for benign and yet protective use in the neonatal and preterm infant population, highlighting it as candidate for future research in well-defined clinical studies.

Overall, DEX exerts neuroprotection by targeting multiple injury pathways, including oxidative stress, neurotrophic depletion, synaptic dysfunction, and mitochondrial impairment [39,40,41,90,91]. By restoring neurotrophic and plasticity signaling, it promotes not only neuronal survival but functional network integration [40,85,94]. Long-term data indicate preserved hippocampal plasticity and intact LTP following neonatal DEX exposure, supporting its safety and translational potential in the preterm brain [75,94].

### 4.5. Limitations and Future Research Directions

For gene expression analyses, we used one complete cerebral hemisphere after removal of the olfactory bulb and cerebellum. We acknowledge that the obtained transcriptional data cannot be attributed exclusively to the hippocampus and that the investigated markers are also expressed in other regions of the developing brain [95,96,97,98,99,100]. The experimental approach was chosen deliberately because both hyperoxia-induced injury and systemic α2-adrenoceptor agonist exposure are not restricted to a single anatomical region but affect multiple cellular and developmental pathways throughout the immature brain. During the early postnatal period, however, the dentate gyrus represents one of the most active neurogenic niches and is therefore particularly susceptible to disturbances in proliferation, differentiation, and neuronal maturation [101]. Consequently, transcriptional alterations detected in whole-hemisphere homogenates are likely to reflect, at least in part, hippocampus-associated developmental processes. In addition, the use of whole-hemisphere tissue reduced variability associated with microdissection of small and developmentally dynamic brain structures in neonatal animals and allowed a robust assessment of global neurodevelopmental responses. Further region-specific transcriptional studies may help to refine these observations in future investigations.

We acknowledge that a limitation of the current study is the lack of immediate functional validation, including detailed structural assessments of spine morphogenesis or long-term behavioral and cognitive phenotypic testing. While neonatal hyperoxia is well-known to cause lasting motor and behavioral deficits, the present study deliberately focused on mapping the longitudinal, multi-stage molecular and cellular landscape (P9 to P14) during the recovery phase, moving beyond simple acute snapshot analyses. Demonstrating that a single dose of dexmedetomidine can partially rescue neural progenitor niches and protect cellular maturation during this critical developmental window provides a necessary mechanistic foundation. Nevertheless, future long-term studies utilizing adolescent and adult behavioral matrices alongside high-resolution structural imaging are warranted to definitively confirm whether these preserved molecular trajectories translate into sustained functional neuroprotection.

Furthermore, the present study utilized a single-dose regimen designed to intercept the immediate, hyperacute oxidative and apoptotic cascades triggered at the onset of the 24 h hyperoxic insult. While our findings demonstrate that this single early intervention is sufficient to yield sustained protective and regenerative effects on the hippocampal neurogenic niche up to P14, we acknowledge that alternative treatment schemes, such as repeated dosing during the recovery phase or delayed post-injury administration, were not evaluated. Investigating repeated or post-injury dosing protocols remains a critical objective for future studies, particularly to optimize therapeutic strategies for clinical scenarios characterized by chronic, prolonged, or intermittent oxygen fluctuations in the neonatal intensive care unit.

## 5. Conclusions

In summary, neonatal hyperoxia disrupts hippocampal development via three key mechanisms: persistent oxidative–apoptotic signaling, depletion of the neural progenitor pool, and suppression of the neurotrophic niche required for maturation. Dexmedetomidine (DEX) counteracts these processes through a multimodal neuroprotective profile. By enhancing antioxidant and autophagic responses, DEX limits apoptotic signaling, while acting as an ontogenetic stabilizer that prevents maturation arrest and maladaptive reactive proliferation. Through preservation of neurogenic timing and neurotrophic support, DEX maintains the structural and functional trajectory of the developing brain. These findings highlight its potential not only as a safe sedative but also as a highly efficient neuroprotective agent in preterm infants exposed to hyperoxic stress.

## Figures and Tables

**Table 1:** Quantification of TUNEL-positive cells in different brain regions at postnatal days P9, P11, and P14. Absolute values are given for the control group (NO) as mean ± SD, while all other groups are expressed as percentage of the control (NO = 100%, mean ± SD).

TUNEL	Region	Abs. Value of NO Control [Mean ± SD]	NO [%, Mean ± SD]	NOD [% of NO, Mean ± SD]	HY [% of NO, Mean ± SD]	[*p* vs. NO]	HYD [% of NO, Mean ± SD]	[*p* vs. HY]
**P9**	**FC**	15 ± 1.1	100 ± 7.4	103 ± 13.7	218 ± 7.8	(*p* > 0.0001)	122 ± 10.7	(*p* > 0.0001)
	**RSC**	13 ± 1.5	100 ± 11.8	117 ± 9.4	171 ± 15.5	(*p* > 0.0001)	120 ± 26.1	(*p* > 0.001)
	**HC**	8 ± 1.3	100 ± 15.9	96 ± 18.9	112 ± 10.8		89 ± 6.5	
	**Th**	6 ± 1.1	100 ± 17.8	99 ± 12.1	122 ± 28.6		96 ± 33.1	
	**HTh**	11 ± 1.3	100 ± 11.8	115 ± 26.5	140 ± 13.4	(*p* > 0.05)	110 ± 26.2	
**P11**	**FC**	15 ± 1.6	100 ± 10.7	97 ± 15.3	184 ± 10.2	(*p* > 0.0001)	105 ± 11.4	(*p* > 0.0001)
	**RSC**	10 ± 1.6	100 ± 16.0	96 ± 23.0	195 ± 22.0	(*p* > 0.0001)	107 ± 17.1	(*p* > 0.0001)
	**HC**	9 ± 1.0	100 ± 11.4	104 ± 11.2	106 ± 15.6		99 ± 10.1	
	**Th**	4 ± 0.9	100 ± 24.6	106 ± 18.6	122 ± 23.8		99 ± 16.0	
	**HTh**	18 ± 1.8	100 ± 10.0	99 ± 12.9	138 ± 21.1	(*p* > 0.001)	94 ± 11.0	
**P14**	**FC**	17 ± 1.6	100 ± 9.3	103 ± 13.3	127 ± 12.7	(*p* > 0.01)	80 ± 9.5	(*p* > 0.0001)
	**RSC**	18 ± 2.0	100 ± 12.0	101 ± 12.4	134 ± 8.6	(*p* > 0.05)	108 ± 23.2	(*p* > 0.05)
	**HC**	10 ± 1.9	100 ± 19.0	98 ± 12.3	98 ± 4.6		95 ± 11.0	(*p* > 0.05)
	**Th**	5 ± 0.8	100 ± 15.2	83 ± 9.9	107 ± 13.8		93 ± 15.1	
	**HTh**	23 ± 4.5	100 ± 19.5	85 ± 11.1	89 ± 19.6		87 ± 21.6	

FC, frontal cortex; RSC, retrosplenial cortex; HC, hippocampus; Th, thalamus; HTh, hypothalamus; NO, normoxia (control); NOD, normoxia + dexmedetomidine; HY, hyperoxia; HYD, hyperoxia + dexmedetomidine.

**Table 2:** Quantification of cCasp3-positive cells in different brain regions at postnatal days P9, P11, and P14. Absolute values are given for the control group (NO) as mean ± SD, while all other groups are expressed as percentage of the control (NO = 100%, mean ± SD).

cCasp3	Region	Abs. Value of NO Control [Mean ± SD]	NO [%, Mean ± SD]	NOD [% of NO, Mean ± SD]	HY [% of NO, Mean ± SD]	[*p* vs. NO]	HYD [% of NO, Mean ± SD]	[*p* vs. HY]
**P9**	**FC**	9.0 ± 1.1	100 ± 12.4	105 ± 22.9	264 ± 29.2	(*p* > 0.0001)	148 ± 40.3	(*p* > 0.0001)
	**RSC**	5.1 ± 1.5	100 ± 30.5	167 ± 50.5	313 ± 42.5	(*p* > 0.0001)	206 ± 51.8	(*p* > 0.01)
	**HC**	4.3 ± 0.9	100 ± 21.0	97 ± 11.0	128 ± 13.6	(*p* > 0.05)	77 ± 13.6	(*p* > 0.0001)
**P11**	**FC**	11.9 ± 1.6	100 ± 13.3	105 ± 19.1	177 ± 28.5	(*p* > 0.0001)	98 ± 14.3	(*p* > 0.0001)
	**RSC**	7.9 ± 1.6	100 ± 20.1	95 ± 28.8	231 ± 27.5	(*p* > 0.0001)	118 ± 18.0	(*p* > 0.0001)
	**HC**	3.6 ± 1.1	100 ± 31.7	129 ± 27.1	159 ± 28.4	(*p* > 0.01)	94 ± 18.2	(*p* > 0.05)
**P14**	**FC**	10.1 ± 1.5	100 ± 15.7	104 ± 22.6	166 ± 21.5	(*p* > 0.0001)	70 ± 13.6	(*p* > 0.0001)
	**RSC**	10.9 ± 2.4	100 ± 24.0	102 ± 21.0	168 ± 14.6	(*p* > 0.001)	112 ± 27.8	(*p* > 0.0001)
	**HC**	3.0 ± 0.7	100 ± 23.6	97 ± 15.8	164 ± 18.3	(*p* > 0.001)	103 ± 23.5	(*p* > 0.001)

FC, frontal cortex; RSC, retrosplenial cortex; HC, hippocampus; NO, normoxia (control); NOD, normoxia + dexmedetomidine; HY, hyperoxia; HYD, hyperoxia + dexmedetomidine.

## Data Availability

The data presented in this study are available on request from the corresponding author due to the ongoing nature of the research project. The oligonucleotides used for qPCR and their target gene sequences are summarized in the Supplementary Table S1.

## Supplementary Materials

The following supporting information can be downloaded at: https://www.mdpi.com/article/10.3390/cells15121094/s1, Table S1: Sequences of oligonucleotides, Table S2: Underlying 2^−ΔΔCt^ values utilized for the generation of RT-qPCR box-and-whisker plots at P9. Table S3: Underlying 2^−ΔΔCt^ values utilized for the generation of RT-qPCR box-and-whisker plots at P11. Table S4: Underlying 2^−ΔΔCt^ values utilized for the generation of RT-qPCR box-and-whisker plots at P14.
