# Supplementary material for: Dexmedetomidine Preserves Hippocampal Neurogenesis During Recovery from Neonatal Hyperoxia in Rats
Source: Cells. 2026 Jun 16;15(12):1094. doi: 10.3390/cells15121094 (PMC13297234; doi:10.3390/cells15121094)
Supplement: Supplementary file 1 [file cells-15-01094-s001.zip › Table S1 Sequences of oligonucleotides.pdf]

Table S1 Sequences of oligonucleotides

	oligonucleotide sequence 5´-3´	Accession No.
AIF		
forward	CACAAAGACACTGCAGTTCAGACA	NM_031356.1
reverse	AGGTCCTGAGCAGAGACATAGAAAG	
probe	6−FAM−AGAAGCATCTATTTCCAGCC−TAMRA	
Ascl1 (Mash1)		
forward	AACTTCAGTGGCTTCGGCTA	NM_022384.1
reverse	GCCCAGGTTAACCAACTTGA	
probe	6−FAM−AGCCTTCCACAGCAGCAG−TAMRA	
Atg5		
forward	ACATCAGCATTGTGCCCCA	NM_001014250.1
reverse	TGTCATGCTTCGGTGTCCTG	
probe	6−FAM−CAGACTGAAGGCCGTGTCCTGCTCA−TAMRA	
Atg12		
forward	TCTGCCTAGCCTGGAACTCAG	NM_001038495.1
reverse	TAGCCCTGTGTGCTCTGCTTT	
probe	6−FAM−CCTGTCCGTGAAGCTCACCCAGC−TAMRA	
Beclin1		
forward	GCAGCACCATGCAGGTGAG	NM_053739.2
reverse	TGGTCACTCGGTCCAGGATC	
probe	6−FAM−TCGTGTGCCAGCGCTGTAGCCA−TAMRA	
BDNF		
forward	TCAGCAGTCAAGTGCCTTTGG	NM_012513.4
reverse	CGCCGAACCCTCATAGACATG	
probe	6−FAM−CCTCCTCTGCTCTTTCTGCTGGAGGAATACAA −TAMRA	
Calb1		
forward	CGACGCTGATGGAAGTGGTT	NM_031984.2
reverse	TCCAATCCAGCCTTCTTTCG	
probe	6−FAM−AAGGAAAGGAGCTGCAGAA−TAMRA	
Casp3		
forward	ACAGTGGAACTGACGATGATATGG	NM_012922.2
reverse	AATAGTAACCGGGTGCGGTAGA	
probe	6−FAM−ATGCCAGAAGATACCAGTGG−TAMRA	
CycD2		
forward	CGTACATGCGCAGGATGGT	NM_199501.1
reverse	AATTCATGGCCAGAGGAAAGAC	
probe	6−FAM−TGGATGCTAGAGGTCTGTGA−TAMRA	
GCLC		
forward	GGAGGACAACATGAGGAAACG	NM_012815.2
reverse	GCTCTGGCAGTGTGAATCCA	
probe	6−FAM−TCAGGCTCTTTGCACGATAA−TAMRA	
GFAP		
forward	TCTGGACCAGCTTACTACCAACAG	NM_017009.2
reverse	TGGTTTCATCTTGGAGCTTCTG	
probe	6−FAM−AGAGGGACAATCTCACACAG−TAMRA	
Hes5		
forward	ATGCTCAGTCCCAAGGAGAA	NM_024383.1
reverse	TAGTCCTGGTGCAGGCTCTT	

probe	6−FAM−CCCAACTCCAAACTGGAGAA−TAMRA	
HPRT		
forward	GGAAAGAACGTCTTGATTGTTGAA	NM_012583.2
reverse	CCAACACTTCGAGAGGTCCTTTT	
probe	6−FAM−CTTTCCTTGGTCAAGCAGTACAGCCCC−TAMRA	
Keap1		
forward	GATCGGCTGCACGGAACT	NM_057152.2
reverse	GCAGTGTGACAGGTTGAAGAACTC	
probe	6−FAM−CTCGGGAGTATATCTACATGC−TAMRA	
NeuN		
forward	GCTGAATGGGACGATCGTAGAG	NM_001134498.2
reverse	CATATGGGTTCCCAGGCTTCT	
probe	6−FAM−AGGTCAATAATGCCACGGC−TAMRA	
NeuroD1		
forward	TCAGCATCAATGGCAACTTC	NM_019218.2
reverse	AAGATTGATCCGTGGCTTTG	
probe	6−FAM−TTACCATGCACTACCCTGCA−TAMRA	
NeuroD2		
forward	TCTGGTGTCCTACGTGCAGA	NM_019326.1
reverse	CCTGCTCCGTGAGGAAGTTA	
probe	6−FAM−TGCCTGCAGCTGAACTCTC−TAMRA	
NGF		
forward	ACCCAAGCTCACCTCAGTGTCT	NM_001277055.1
reverse	GACATTACGCTATGCACCTCAGAGT	
probe	6−FAM−CAATAAAGGCTTTGCCAAGG−TAMRA	
Nrf2		
forward	ACTCCCAGGTTGCCCACAT	NM_031789.2
reverse	GCGACTCATGGTCATCTACAAATG	
probe	6−FAM−CTTTGAAGACTGTATGCAGC−TAMRA	
Nrg1		
forward	GGGACCAGCCATCTCATAAA	NM_001271118
reverse	ATCTTGACGGGTTTGACAGG	
probe	6−FAM−ACTTTCTGTGTGAATGGGGG−TAMRA	
Nrp1		
forward	TGAGCCCTGTGGTCTATTCC	NM_145098
reverse	CCTCTGGCTTCTGGTAGTGC	
probe	6−FAM−TGTGGGTACACTGAGGGTCA−TAMRA	
NT3		
forward	AGAACATCACCACGGAGGAAA	NM_031073.3
reverse	GGTCACCCACAGGCTCTCA	
probe	6−FAM−AGAGCATAAGAGTCACCGAG−TAMRA	
Pax6		
forward	TCCCTATCAGCAGCAGTTTCAGT	NM_013001.2
reverse	GTCTGTGCGGCCCAACAT	
probe	6−FAM−CTCCTCCTTTACATCGGGTT−TAMRA	
Prox1		
forward	TGCCTTTTCCAGGAGCAACTAT	NM_001107201.1
reverse	CCGCTGGCTTGGAAACTG	
probe	6−FAM−ACATGAACAAAAACGGTGGC−TAMRA	
Scl1a3		

forward	CCCTGCCCATCACTTTCAAG	NM_001289942.1
reverse	GCGGTCCCATCCATGTTAA	
probe	6–FAM–CTGGAAGAAAACAATGGTGTGG–TAMRA	
Sema3a		
forward	GAAAACGGTCGTGGGAAGAG	NM_017310
reverse	AGCAAAGTCTCGTCCCATGA	
probe	6–FAM–GACCCCAAACTTCTGACTGC–TAMRA	
Sema3f		
forward	CCATGCGCACAGATCAGTAC	NM_001108185
reverse	AGTTTATCGTCGTTGCGCTC	
probe	6–FAM–CGGTGGCTCAATGATCCTTC–TAMRA	
SOD1		
forward	CAGAAGGCAAGCGGTGAAC	NM_017050.1
reverse	CCCCATATTGATGGACATGGA	
probe	6–FAM–TACAGGATTAACTGAAGGCG–TAMRA	
SOD2		
forward	GACCTACGTGAACAATCTGAACGT	NM_017051.2
reverse	AGGCTGAAGAGCAACCTGAGTT	
probe	6–FAM–ACCGAGGAGAAGTACCACGA–TAMRA	
SOD3		
forward	GGAGAGTCCGGTGTCGACTTAG	NM_012880.1
reverse	CTCCATCCAGATCTCCAGGTCTT	
probe	6–FAM–CTGGTTGAGAAGATAGGCGA–TAMRA	
Sox2		
forward	ACAGATGCAGCCGATGCA	NM_001109181.1
reverse	GGTGCCCTGCTGCGAGTA	
probe	6–FAM–CAGTACAACTCCATGACCAG–TAMRA	
Syp		
forward	TTCAGGCTGCACCAAGTGTA	NM_012664
reverse	TTCAGCCGACGAGGAGTAGT	
probe	6–FAM–AGGGGGCACTACCAAGATCT–TAMRA	
Tbr1		
forward	TCCCAATCACTGGAGGTTTCA	NM_001191070.1
reverse	GGATGCATATAGACCCGGTTTC	
probe	6–FAM–AAATGGGTTCCTTGTGGCAA–TAMRA	
Tbr2		
forward	ACGCAGATGATAGTGTTGCAGTCT	XM_006226608.2
reverse	ATTCAAGTCCTCCACACCATCCT	
probe	6–FAM–CACAAATACCAACCTCGACT–TAMRA	

Abbreviations: AIF: Apoptosis-Inducing Factor, Ascl1: Achaete-Scute Family bHLH Transcription Factor 1, Atg5/12: Autophagy Related 5/12, Beclin1: Beclin 1, autophagy related, BDNF: Brain-Derived Neurotrophic Factor, Calb1: Calbindin 1, Casp3: Caspase 3, CycD2: Cyclin D2, GFAP: Glial Fibrillary Acidic Protein, Hes5: Hes Family bHLH Transcription Factor 5, HPRT: Hypoxanthine Phosphoribosyltransferase, Keap1: Kelch-Like ECH-Associated Protein 1, NeuN: RNA Binding Fox-1 Homolog 3 (RBFOX3), NeuroD1/2: Neuronal Differentiation ½, NGF: Nerve Growth Factor, Nrf2: Nuclear Factor, Erythroid 2–Like 2, Nrg1: Neuregulin 1, Nrp1: Neuropilin 1, NT3: Neurotrophin 3, Pax6: Paired Box 6, Prox1: Prospero Homeobox 1, Slc1a3: Solute Carrier Family 1 Member 3 (Glutamat-Transporter EAAT1/GLAST), Sema3a/f: Semaphorin 3A/F, SOD: Superoxide

Dismutase, Sox2: SRY-Box Transcription Factor 2, Syp: Synaptophysin, Tbr1/2: T-Box Brain 1/2
