# Supplementary material for: Dexmedetomidine Preserves Hippocampal Neurogenesis During Recovery from Neonatal Hyperoxia in Rats
Source: Cells. 2026 Jun 16;15(12):1094. doi: 10.3390/cells15121094 (PMC13297234; doi:10.3390/cells15121094)
Supplement: Supplementary file 1 [file cells-15-01094-s001.zip › Table S2 2^-ddCt data P9.pdf]

Supplementary Table S2. Underlying $2^{-\Delta\Delta Ct}$ values utilized for the generation of RT-qPCR box-and-whisker plots at P9

			Ascl1	Atg5	Atg12	AIF	BDNF	Beclin1	Casp3	Calb1	CycD2	GCLC
**sample**	**treatment**		**2^(-ddCT)**	**2^(-ddCT)**	**2^(-ddCT)**	**2^(-ddCT)**	**2^(-ddCT)**	**2^(-ddCT)**	**2^(-ddCT)**	**2^(-ddCT)**	**2^(-ddCT)**	**2^(-ddCT)**
1	NaCl	21% oxygen	1,008368007	0,827929091	0,914238911	0,907291138	0,846147966	0,966573781	0,768657313	0,874855388	0,805545295	0,985595178
2	NaCl	21% oxygen	1,206254323	1,024482218	1,037845862	0,968285968	0,86904019	1,029058589	0,865922818	1,195215032	1,008995769	0,787570211
3	NaCl	21% oxygen	0,905771652	1,050755514	0,923144938	1,03606621	0,943707806	1,052831973	1,136304119	0,895779014	1,04373806	0,94897035
4	NaCl	21% oxygen	1,008548441	1,109487522	1,176733931	1,172762133	1,123982505	1,247263383	1,141322509	1,189871948	0,984670114	0,994756965
5	NaCl	21% oxygen	0,826532549	0,998542816	1,002861189	0,981330251	0,977534455	0,956641925	1,027442063	1,071038799	0,973007422	1,01677273
6	NaCl	21% oxygen	1,088846379	1,012771895	0,967427542	0,954634359	1,311549394	0,998197349	1,127529072	0,837744084	0,991088014	1,057080127
7	DEX **5**µg/kg	21% oxygen	1,163957388	0,965382764	0,874691162	0,935281654	0,975566862	0,86242425	1,01461706	1,181323754	0,930052928	1,019842689
8	DEX **5**µg/kg	21% oxygen	0,936647979	0,862609437	0,918599031	1,033652321	0,809116098	0,980389647	1,177678525	1,163225166	0,788161432	0,78974792
9	DEX **5**µg/kg	21% oxygen	0,988692543	0,832496201	0,751972801	1,010038684	0,85979893	0,857294279	1,00329766	1,313135524	0,819689264	0,779101416
10	DEX **5**µg/kg	21% oxygen	0,917706813	0,963840092	0,934950575	1,21514445	0,859427682	1,072237571	1,323967368	1,240940607	0,977467139	0,896197547
11	DEX **5**µg/kg	21% oxygen	0,95405882	0,878659891	0,914232545	0,852619318	1,129747017	0,768004087	1,072510639	1,235022412	0,95768279	0,867400881
12	DEX **5**µg/kg	21% oxygen	0,846096546	0,998709608	0,851822968	0,988538518	0,881175857	0,995716341	1,296807903	1,318112382	0,818772614	0,726836287
13	NaCl	80% oxygen, 24h	0,442973269	0,895350235	0,70909476	1,012550917	0,627298165	0,693368492	1,514601624	0,748345921	0,706638142	1,08526469
14	NaCl	80% oxygen, 24h	0,572301266	0,932372625	0,84652667	0,939313514	0,729689195	0,692220166	1,523860405	0,843176106	0,584607621	0,777988681
15	NaCl	80% oxygen, 24h	0,635239598	0,956934633	0,716526047	0,970710178	0,77308763	0,752001688	1,44422938	0,978130319	0,665397235	0,750623399
16	NaCl	80% oxygen, 24h	0,684222349	1,089087112	0,770347184	1,153734637	0,738132838	0,717867214	1,330742284	0,862599109	0,657878976	0,962244672
17	NaCl	80% oxygen, 24h	0,537873674	0,816271445	0,594567301	1,030400923	0,662861513	0,533122165	1,413965175	0,797430691	0,757645968	1,070620392
18	NaCl	80% oxygen, 24h	0,340885092	0,775981199	0,422601118	0,743873282	0,566985426	0,301196404	1,281711808	0,613290727	0,382496755	0,799223672
19	DEX **5**µg/kg	80% oxygen, 24h	0,857102667	1,059858629	1,133937154	0,983981367	1,016151997	0,995672462	1,10914464	1,326811181	0,932665059	0,595987513
20	DEX **5**µg/kg	80% oxygen, 24h	0,729895638	0,822516634	0,92767393	0,781635566	1,097233238	0,964281429	1,087787517	1,278097435	0,985600477	0,54259399
21	DEX **5**µg/kg	80% oxygen, 24h	0,656834919	1,091268752	1,160821913	1,133290506	0,918778883	1,234864132	1,122463343	1,504330062	0,869061535	0,68884645
22	DEX **5**µg/kg	80% oxygen, 24h	0,67078275	1,024474543	0,988850311	1,139385412	0,952482319	1,26942952	1,087701232	1,205889746	0,932644097	0,556497524
23	DEX **5**µg/kg	80% oxygen, 24h	1,169316737	1,450527714	0,88583608	0,97656788	0,788506157	1,409725829	1,163541592	1,344573941	0,891255909	0,714794195
24	DEX **5**µg/kg	80% oxygen, 24h	1,000398273	1,013927629	0,917421684	0,997252034	1,100598514	0,979755172	1,193142912	1,390917627	0,779662503	0,745701067

Supplementary Table S2. Underlying $2^{-\Delta\Delta Ct}$ values utilized for the generation of RT-qPCR box-and-whisker plots at P9

			**GFAP**	**Hes5**	**Keap1**	**NeuN**	**NeuroD1**	**NeuroD2**	**NGF**	**Nrf2**	**Nrg1**	**Nrp1**
**sample**	**treatment**		**2^(-ddCT)**	**2^(-ddCT)**	**2^(-ddCT)**	**2^(-ddCT)**	**2^(-ddCT)**	**2^(-ddCT)**	**2^(-ddCT)**	**2^(-ddCT)**	**2^(-ddCT)**	**2^(-ddCT)**
1	NaCl	21% oxygen	1,00486191	0,826013237	0,884695643	0,895567013	0,732408967	0,920597524	0,745583298	0,88959764	0,917883921	1,011179045
2	NaCl	21% oxygen	0,812170406	0,861262498	0,765407478	0,928649759	0,727035675	0,927259972	0,806770752	0,769363657	0,758607724	1,179445834
3	NaCl	21% oxygen	1,213705601	1,099579627	1,202290985	1,051731323	1,150093943	1,119261641	1,193747946	1,209231308	0,820760594	1,118508457
4	NaCl	21% oxygen	1,046406571	1,135161502	1,132711597	1,142367615	1,041573041	1,02322013	1,307082888	1,209411441	1,121525184	0,868999391
5	NaCl	21% oxygen	0,953155865	1,044844129	1,144923403	0,957811071	1,210994422	0,955781873	0,929949073	1,206050902	1,301878139	0,81874156
6	NaCl	21% oxygen	1,012205555	1,077808516	0,947126614	1,044863161	1,294569694	1,070210481	1,14572068	0,828372394	1,198392591	1,053630558
7	DEX **5**µg/kg	21% oxygen	0,945792879	0,879908858	0,939553455	0,943283478	0,897300441	0,999902539	0,853362471	1,022953143	1,067393935	0,709488202
8	DEX **5**µg/kg	21% oxygen	0,887824289	0,939594778	0,800709478	0,89961311	0,730666943	1,07468475	0,867114771	0,820055729	0,82609043	0,643290714
9	DEX **5**µg/kg	21% oxygen	0,754959982	0,894201187	0,204374747	0,803590763	1,094727268	0,933081197	0,824038917	0,939674477	0,863018208	0,835045221
10	DEX **5**µg/kg	21% oxygen	1,092910731	1,016252012	1,180399515	0,967580992	0,984544975	1,232177128	0,863731896	1,234933174	1,039173455	0,805950709
11	DEX **5**µg/kg	21% oxygen	1,051327887	0,807573313	0,944404973	0,754550668	0,667999901	0,789476998	0,713054787	1,153169	1,386777471	0,601301533
12	DEX **5**µg/kg	21% oxygen	0,822351265	0,843675497	0,902774113	0,972239124	0,671913052	1,174463206	0,993555827	0,873907946	0,935827616	0,554104188
13	NaCl	80% oxygen, 24h	0,869977409	0,720999071	1,207760305	0,753634058	0,399193101	0,854600272	0,682670335	0,895972166	1,227153274	0,623542745
14	NaCl	80% oxygen, 24h	0,869183768	0,750426074	0,899231514	0,507955254	0,723385984	1,046787128	0,612405137	0,840136585	0,815007655	0,726329967
15	NaCl	80% oxygen, 24h	0,604515229	0,879930185	0,774070313	0,439550586	0,767709892	0,927233411	0,614347308	0,784767741	0,905691297	0,719018455
16	NaCl	80% oxygen, 24h	0,952865657	0,710023645	1,097465717	0,726133524	0,401548566	1,097559757	0,795226668	1,508168917	1,214249962	0,686703177
17	NaCl	80% oxygen, 24h	0,728083469	0,676700653	1,232923757	0,623809128	0,577625639	1,272330453	0,697768912	1,242025107	0,976350663	0,60334695
18	NaCl	80% oxygen, 24h	0,688359435	0,477924459	0,642499312	0,309452187	0,709970642	0,809854809	0,475624051	0,646756167	0,689562469	0,399281341
19	DEX **5**µg/kg	80% oxygen, 24h	0,79019086	1,042396926	0,906281144	0,916204782	1,175899095	0,827872114	0,926752163	1,163452913	1,393670364	1,121842082
20	DEX **5**µg/kg	80% oxygen, 24h	0,616540758	0,261897663	0,682055981	0,931893871	0,855576098	0,767426374	0,769070478	0,693150009	1,403003202	0,909426568
21	DEX **5**µg/kg	80% oxygen, 24h	0,571331936	0,922884514	1,100844804	1,134821146	0,70827974	1,03993338	1,084595324	0,906190993	1,442541481	0,963365983
22	DEX **5**µg/kg	80% oxygen, 24h	0,598104078	0,830463638	0,680782729	1,277605872	0,78702563	0,965039853	0,985098155	0,78244265	1,390500516	0,844310523
23	DEX **5**µg/kg	80% oxygen, 24h	0,759516152	0,879354834	1,188875737	1,389097271	0,99859844	1,017672894	1,000833165	1,335977822	1,476568475	1,281320388
24	DEX **5**µg/kg	80% oxygen, 24h	0,672686255	1,115977569	0,937381364	0,886674662	0,853268308	0,239937944	0,935432507	1,153302459	1,452743106	1,064069358

Supplementary Table S2. Underlying $2^{-\Delta\Delta Ct}$ values utilized for the generation of RT-qPCR box-and-whisker plots at P9

			**NT3**	**Pax6**	**Prox1**	**Scl1a3**	**Sema3a**	**Sema3f**	**SOD1**	**SOD2**	**SOD3**	**Sox2**
**sample**	**treatment**		**2^(-ddCT)**	**2^(-ddCT)**	**2^(-ddCT)**	**2^(-ddCT)**	**2^(-ddCT)**	**2^(-ddCT)**	**2^(-ddCT)**	**2^(-ddCT)**	**2^(-ddCT)**	**2^(-ddCT)**
1	NaCl	21% oxygen	0,867540199	0,897113908	0,826131672	0,899396554	0,836683554	0,875541495	1,267226102	1,150619905	1,263575276	0,776604058
2	NaCl	21% oxygen	0,92408379	0,964954629	1,196995251	1,054089179	1,258644654	0,976568956	1,049620553	0,860447419	0,932949882	0,945051234
3	NaCl	21% oxygen	1,034344946	0,800505913	1,152707282	1,214223198	0,930202377	0,929864791	1,056468772	1,199965707	1,01722919	0,951617606
4	NaCl	21% oxygen	1,121339157	1,113744192	0,950167136	0,853062433	1,228723098	1,193374667	0,9963769	1,042028802	0,827824699	1,215209158
5	NaCl	21% oxygen	0,871965683	1,129650716	0,84462015	1,093397339	0,923028499	1,125386979	0,909417483	0,900384684	1,231215418	1,093522547
6	NaCl	21% oxygen	1,233381025	1,146968096	1,09314512	0,931352468	0,900096461	0,936530333	0,785362119	0,897154261	0,818180882	1,077466426
7	DEX **5**µg/kg	21% oxygen	0,948495571	1,084115276	0,90731173	1,190663794	1,038985688	0,949974121	1,231246671	1,038742358	0,995505328	0,920516644
8	DEX **5**µg/kg	21% oxygen	0,891343435	1,000621229	0,675192032	0,947700045	0,819772207	1,022826324	0,783890489	0,80164904	0,850687055	0,828857258
9	DEX **5**µg/kg	21% oxygen	0,944020921	0,993918185	1,055336477	0,176236777	1,03429944	0,901059019	0,941878861	0,990645628	1,133365939	0,897596774
10	DEX **5**µg/kg	21% oxygen	1,077442099	1,164854807	1,031068422	1,194294574	0,737043709	1,145235733	1,06555564	1,115108157	1,200182311	1,015461173
11	DEX **5**µg/kg	21% oxygen	0,791548277	1,104651333	0,756691561	0,984284523	0,919215601	0,778959399	1,412229133	1,088852681	1,079909913	0,719042009
12	DEX **5**µg/kg	21% oxygen	0,993802207	0,96133315	0,696302428	0,832213695	1,180882143	1,209236992	0,836668314	0,870885664	0,869342489	0,889240878
13	NaCl	80% oxygen, 24h	0,432325249	1,192849872	0,704131685	0,879258901	0,889119613	1,24106962	0,911852624	0,94334765	0,688668723	0,617287726
14	NaCl	80% oxygen, 24h	0,548832361	0,745453863	0,779187989	0,586653911	1,086402221	1,056824377	0,759708546	0,704316951	0,504997762	0,611611085
15	NaCl	80% oxygen, 24h	0,722760481	0,959920466	0,696617454	0,643159782	0,958936062	0,911600969	0,866331326	0,973649498	0,680240658	0,63424878
16	NaCl	80% oxygen, 24h	0,72766336	1,116401205	0,768999963	0,871579426	0,900671412	1,356305542	1,043544923	1,019856128	1,716301663	0,772027916
17	NaCl	80% oxygen, 24h	0,586218289	1,181800615	0,691989624	0,851475051	0,834752665	1,139578492	1,321241944	1,012611902	0,637787626	0,691150148
18	NaCl	80% oxygen, 24h	0,435037801	0,62300881	0,569108466	0,367288421	0,814921441	0,697246644	0,835569389	0,750915116	0,382480787	0,242288513
19	DEX **5**µg/kg	80% oxygen, 24h	0,89388356	1,250540582	1,208778785	0,86158853	1,327225203	1,022057182	1,064919079	0,833748732	0,929021239	0,96499887
20	DEX **5**µg/kg	80% oxygen, 24h	1,105220365	1,286956203	1,107628823	0,859189171	1,241001293	0,616127255	0,737903545	0,632154604	0,820008099	0,721354792
21	DEX **5**µg/kg	80% oxygen, 24h	0,66271733	1,192150924	0,718845151	1,224835876	1,832140408	1,433482675	0,849226688	0,876846682	0,981506294	0,954296444
22	DEX **5**µg/kg	80% oxygen, 24h	0,924419657	1,163688567	0,75653018	0,814441727	1,407927454	1,155627842	0,831362918	0,823817173	0,839871238	0,972847866
23	DEX **5**µg/kg	80% oxygen, 24h	1,023782407	0,986912914	0,644814303	1,099109609	1,51814843	1,261483527	1,114293372	1,298518295	1,113645657	0,906627108
24	DEX **5**µg/kg	80% oxygen, 24h	0,908730325	1,1920385	1,164459653	1,323270442	1,523764499	1,086082611	1,201195389	1,126384359	0,978225601	0,947646018

Supplementary Table S2. Underlying $2^{-\Delta\Delta Ct}$ values utilized for the generation of RT-qPCR box-and-whisker plots at P9

sample	treatment		Syp 2^(-ddCT)	Tbr1 2^(-ddCT)	Tbr2 2^(-ddCT)	TNFa 2^(-ddCT)
1	NaCl	21% oxygen	0,845309218	0,959015513	0,985464222	0,987014691
2	NaCl	21% oxygen	0,838034093	0,95229443	1,231362146	0,985535603
3	NaCl	21% oxygen	1,175723142	1,114287054	0,872418569	0,951949623
4	NaCl	21% oxygen	1,142806943	1,155780465	1,175062382	0,879216202
5	NaCl	21% oxygen	1,005175529	0,926716306	0,752614449	1,129064727
6	NaCl	21% oxygen	1,045208334	0,917453125	1,068107499	1,087866433
7	DEX **5**µg/kg	21% oxygen	0,901076293	1,03790613	1,395507897	1,227593877
8	DEX **5**µg/kg	21% oxygen	0,864477141	0,910457815	1,432074167	0,759259438
9	DEX **5**µg/kg	21% oxygen	0,998566505	0,841200342	1,588739833	0,782175659
10	DEX **5**µg/kg	21% oxygen	1,055030733	0,951413535	1,754310952	1,158612936
11	DEX **5**µg/kg	21% oxygen	0,734983679	0,859897577	1,862601784	2,814661434
12	DEX **5**µg/kg	21% oxygen	1,026602227	1,083786993	1,301444719	1,088140692
13	NaCl	80% oxygen, 24h	0,826719671	1,193133544	0,624825294	1,150659322
14	NaCl	80% oxygen, 24h	0,668560001	0,774149731	0,672570856	0,893696622
15	NaCl	80% oxygen, 24h	0,616506823	0,773048565	0,665598124	0,893888445
16	NaCl	80% oxygen, 24h	0,946730774	1,186171477	0,764049846	1,173157158
17	NaCl	80% oxygen, 24h	0,726967765	1,097765423	0,514957257	1,009598294
18	NaCl	80% oxygen, 24h	0,407179769	0,707343836	0,571630265	0,873010132
19	DEX **5**µg/kg	80% oxygen, 24h	1,105955585	0,816069378	1,12911249	0,828624163
20	DEX **5**µg/kg	80% oxygen, 24h	0,722846421	0,810858724	0,763979014	0,683938628
21	DEX **5**µg/kg	80% oxygen, 24h	1,132301565	1,136711851	1,460777308	0,97872268
22	DEX **5**µg/kg	80% oxygen, 24h	1,194890511	1,118516673	1,284084628	0,498891841
23	DEX **5**µg/kg	80% oxygen, 24h	1,287000215	1,094404951	1,263897921	0,885020093
24	DEX **5**µg/kg	80% oxygen, 24h	1,20270085	0,871030174	1,433624873	0,930125818
