# Supplementary material for: Dexmedetomidine Preserves Hippocampal Neurogenesis During Recovery from Neonatal Hyperoxia in Rats
Source: Cells. 2026 Jun 16;15(12):1094. doi: 10.3390/cells15121094 (PMC13297234; doi:10.3390/cells15121094)
Supplement: Supplementary file 1 [file cells-15-01094-s001.zip › Table S3 2^-ddCt data P11.pdf]

Supplementary Table S3. Underlying $2^{-\Delta\Delta Ct}$ values utilized for the generation of RT-qPCR box-and-whisker plots at P11

			Ascl1	Atg5	Atg12	AIF	BDNF	Beclin1	Casp3	Calb1	CycD2	Gclc
**sample**	**treatment**		**2^(-ddCT)**	**2^(-ddCT)**	**2^(-ddCT)**	**2^(-ddCT)**	**2^(-ddCT)**	**2^(-ddCT)**	**2^(-ddCT)**	**2^(-ddCT)**	**2^(-ddCT)**	**2^(-ddCT)**
1	NaCl	21% oxygen	1,170755467	1,080731545	0,921600079	1,023032323	0,844885235	1,076619599	1,15806595	0,950144929	1,167335308	0,893079524
2	NaCl	21% oxygen	0,841644878	0,973724661	1,072529096	0,950862552	1,15948697	0,924007518	0,896039937	0,86951423	0,82379251	1,001748353
3	NaCl	21% oxygen	1,082755183	0,993024359	1,115382975	1,051101707	0,971009487	1,051297216	1,005388917	0,996176181	0,907971646	1,179756439
4	NaCl	21% oxygen	0,875624957	1,074789816	1,143466294	0,877841578	1,204531003	0,841488556	0,915981595	0,889501452	0,916730161	0,945729152
5	NaCl	21% oxygen	0,903501281	0,941176396	0,918457855	1,206066227	0,855756269	1,16870315	1,093758144	1,186902016	1,098168563	0,951837451
6	NaCl	21% oxygen	1,184752804	0,946000858	0,863658004	0,923763622	1,019870105	0,972264146	0,9567472	1,150895175	1,137637044	1,052517461
7	DEX 5µg/kg	21% oxygen	1,041108089	1,071942693	1,024207604	1,083440061	0,973277944	1,137513402	1,162802671	1,187847033	1,049135396	0,943606405
8	DEX 5µg/kg	21% oxygen	0,728571308	0,938325046	1,162394108	0,858275123	1,12088885	0,885941919	1,015590077	0,847018004	0,881513339	0,796556976
9	DEX 5µg/kg	21% oxygen	0,978699993	0,952751418	0,965140579	1,041017188	0,798770012	1,153664831	1,151891421	1,084265335	1,110529146	1,036785124
10	DEX 5µg/kg	21% oxygen	0,971711194	0,881401271	0,968297845	0,939351874	0,737876468	0,918448896	1,05870676	0,820046923	0,978459585	0,728253256
11	DEX 5µg/kg	21% oxygen	1,021959583	0,975564713	1,176260999	0,992565769	1,060164324	0,959015739	1,134620605	0,890358767	1,062732744	0,744272081
12	DEX 5µg/kg	21% oxygen	0,804773559	0,968233412	1,225518251	0,903087311	1,027519484	0,917118631	0,96173963	0,816625238	0,819484749	0,829773264
13	NaCl	80% oxygen, 24h	0,671356791	0,872450699	1,062919861	1,008976605	0,804422089	1,089965552	1,464547416	0,742303925	1,574412125	0,986224931
14	NaCl	80% oxygen, 24h	0,604136494	1,018310038	1,088406594	0,894654676	0,849122404	0,955767847	1,485276778	0,620507182	1,358280524	0,844047317
15	NaCl	80% oxygen, 24h	0,691347659	1,001567738	1,212590021	1,20359621	0,823002858	0,980793581	1,358336273	0,771216236	1,391715282	0,755975113
16	NaCl	80% oxygen, 24h	0,733156175	0,827213786	0,918696287	0,942322614	0,790160449	0,770438477	1,294310996	0,701075558	1,39661878	0,743172147
17	NaCl	80% oxygen, 24h	0,665122247	0,894551055	0,914852098	1,021335053	0,647957423	1,019760955	1,328572962	0,652255221	1,280969119	1,18625754
18	NaCl	80% oxygen, 24h	0,658330548	1,034643935	0,935740856	1,189798678	0,671033719	1,047287631	1,35149663	0,787922814	1,328141916	1,049573451
19	DEX 5µg/kg	80% oxygen, 24h	1,266356867	1,027306349	1,046350282	1,179635653	0,984939972	1,048538201	1,009961534	0,934564533	1,046139988	1,280231665
20	DEX 5µg/kg	80% oxygen, 24h	1,021815925	0,973853833	1,031506692	1,230063198	0,976178549	1,119636918	1,082315362	1,35311332	1,120560592	1,278418188
21	DEX 5µg/kg	80% oxygen, 24h	0,893913736	0,974396024	1,151927954	1,022956674	0,972511518	0,954877849	1,012369172	1,031948013	0,947920308	1,363257652
22	DEX 5µg/kg	80% oxygen, 24h	0,922390348	1,09459869	1,077592263	0,986874757	1,08186887	0,924429068	0,995524471	0,989633655	0,94446148	1,188619163
23	DEX 5µg/kg	80% oxygen, 24h	1,165922224	0,864741128	0,900245487	1,145694677	1,068293678	1,163290835	1,149591018	0,841613228	0,938460481	1,80036019
24	DEX 5µg/kg	80% oxygen, 24h	1,231739432	0,972000731	1,202438431	1,081279314	0,956906294	1,281983478	1,092325202	0,89983482	1,166243161	1,618732019

Supplementary Table S3. Underlying $2^{-\Delta\Delta Ct}$ values utilized for the generation of RT-qPCR box-and-whisker plots at P11

			GFAP	Hes5	Keap1	NeuN	NeuroD1	NeuroD2	NGF	Nrf2	Nrg1	Nrp1
**sample**	**treatment**		**2^(-ddCT)**	**2^(-ddCT)**	**2^(-ddCT)**	**2^(-ddCT)**	**2^(-ddCT)**	**2^(-ddCT)**	**2^(-ddCT)**	**2^(-ddCT)**	**2^(-ddCT)**	**2^(-ddCT)**
1	NaCl	21% oxygen	1,143536374	0,907035759	0,914911163	1,231622062	1,251220606	0,974392045	1,166297059	0,87172811	1,040389402	1,142829237
2	NaCl	21% oxygen	0,832528389	0,825330563	0,860806841	0,88047976	0,826604915	0,853933761	0,74187379	0,990361463	0,979513354	0,819876728
3	NaCl	21% oxygen	1,183411376	1,153825754	1,226580769	0,918993315	1,085019383	1,186000555	0,84211606	1,201339294	0,844171128	1,18466471
4	NaCl	21% oxygen	0,971731791	0,904972264	0,851937745	0,893834071	0,961089836	0,85344506	0,807098777	1,026855912	1,1082901	1,060083267
5	NaCl	21% oxygen	0,840236358	1,125309465	1,104073121	1,110058753	0,968402041	1,294243634	1,31559922	0,834541278	1,127890992	0,828747477
6	NaCl	21% oxygen	1,087094612	1,13684222	1,10055947	1,011318473	0,957438642	0,917414474	1,292523788	1,125128703	0,929913693	1,025445181
7	DEX **5**µg/kg	21% oxygen	0,862680805	1,045531367	0,979987218	1,19791223	1,016625068	2,041504606	1,223497967	0,919573163	1,263677072	1,065581201
8	DEX **5**µg/kg	21% oxygen	0,991196396	0,780398282	0,805814984	0,844726933	0,744822396	0,827286966	0,937859865	1,014204031	1,139891072	0,752768542
9	DEX **5**µg/kg	21% oxygen	1,122568768	0,935116471	1,066193287	1,169794748	1,209653525	1,049576488	1,166192623	1,160777441	1,022744892	1,028570787
10	DEX **5**µg/kg	21% oxygen	0,892236851	1,0546845	0,853755692	1,121086355	0,641331623	1,063161561	0,964497002	1,167128593	1,04633359	0,881148921
11	DEX **5**µg/kg	21% oxygen	0,974385747	1,27624941	1,004010008	1,080731323	0,913567931	1,153176793	0,951471412	1,236976701	1,295555256	0,922958903
12	DEX **5**µg/kg	21% oxygen	0,840685115	0,747489158	0,847114638	0,97797419	0,634650551	0,960984196	0,868869386	1,080770044	1,217903188	0,752813162
13	NaCl	80% oxygen, 24h	1,331513753	1,034963322	1,277173895	1,154118862	0,582076282	1,55289105	1,138163914	1,316039148	1,270889828	0,818673141
14	NaCl	80% oxygen, 24h	1,18902042	0,946710831	1,169054784	1,019122409	0,685777741	1,354635409	1,068990592	1,15887912	1,094191155	0,750822199
15	NaCl	80% oxygen, 24h	1,090942133	1,370961575	0,79533292	1,075453384	0,540197478	1,260498961	0,77790744	0,94464602	1,218909952	0,671694473
16	NaCl	80% oxygen, 24h	0,809018285	1,000177629	0,773591509	0,999737911	0,55634511	1,345931646	0,901974575	0,903328521	1,110466652	0,666583281
17	NaCl	80% oxygen, 24h	1,39639622	1,153037369	1,261722393	1,136386188	0,544380117	1,488640967	1,160109727	1,201445924	1,227861714	0,677291229
18	NaCl	80% oxygen, 24h	1,704561609	1,285407568	1,241053559	1,094407845	0,504169443	1,216384004	1,19479889	1,235314316	1,083465924	0,719200538
19	DEX **5**µg/kg	80% oxygen, 24h	1,275064748	1,073111737	1,1926974	1,387780195	1,432271226	1,454546514	1,1748966	1,307813197	1,405863123	1,139548946
20	DEX **5**µg/kg	80% oxygen, 24h	1,220499413	1,156828674	1,446462243	1,339810522	1,233233191	1,422058916	1,233966665	1,232958042	1,28951684	1,015205426
21	DEX **5**µg/kg	80% oxygen, 24h	1,280624004	1,147383052	1,505354484	1,022008479	0,8294027	1,087899018	0,839107638	1,791750736	1,35414912	0,927501615
22	DEX **5**µg/kg	80% oxygen, 24h	1,328046847	1,040558767	1,485928633	1,015188112	1,025775275	1,232999827	1,085912472	1,910278382	1,404825757	1,070140241
23	DEX **5**µg/kg	80% oxygen, 24h	1,395445783	0,920664548	0,995132626	1,041391472	0,939399619	1,481150292	0,874650072	1,214952576	1,37233641	1,015346364
24	DEX **5**µg/kg	80% oxygen, 24h	1,174856163	1,318865767	0,986825991	1,111346174	0,726407336	1,181519498	1,046655663	1,340489171	1,330562756	1,211514386

Supplementary Table S3. Underlying $2^{-\Delta\Delta Ct}$ values utilized for the generation of RT-qPCR box-and-whisker plots at P11

			**NT3**	**Pax6**	**Prox1**	**Scl1a3**	**Sema3a**	**Sema3f**	**SOD1**	**SOD2**	**SOD3**	**Sox2**
**sample**	**treatment**		**2^(-ddCT)**	**2^(-ddCT)**	**2^(-ddCT)**	**2^(-ddCT)**	**2^(-ddCT)**	**2^(-ddCT)**	**2^(-ddCT)**	**2^(-ddCT)**	**2^(-ddCT)**	**2^(-ddCT)**
1	NaCl	21% oxygen	0,912909242	1,181842992	1,23762386	0,967468514	1,244592929	1,062624158	1,171783415	1,026817372	1,179157847	1,073203943
2	NaCl	21% oxygen	1,09055385	1,181225972	0,931452511	0,992184781	0,82719867	0,784724731	0,964862738	0,893748257	0,909043293	0,90539956
3	NaCl	21% oxygen	0,907920347	0,986926991	1,092377128	1,099181056	0,962519667	1,04203422	1,168076415	1,180787087	0,996205376	1,04492333
4	NaCl	21% oxygen	0,99505781	2,040756395	0,830961359	0,964898031	0,807040223	0,93042635	0,753357586	0,917833394	0,873485311	0,812630196
5	NaCl	21% oxygen	0,298084378	0,797541454	0,833759339	0,862205184	1,03369713	1,204985104	0,881990191	0,913031864	1,079702501	1,039991203
6	NaCl	21% oxygen	1,111806527	0,910057715	1,146189617	1,139224495	1,209663956	1,026496126	1,139596069	1,101210138	0,992967179	1,165387658
7	DEX 5µg/kg	21% oxygen	0,984761401	1,125041489	1,120945246	1,110988772	0,891624716	1,375490814	0,901708096	0,84031938	0,973479555	1,222686314
8	DEX 5µg/kg	21% oxygen	1,129466897	1,266422591	1,014189311	1,041790421	0,455965199	0,836906769	0,917975782	0,829125726	1,009936832	1,08971699
9	DEX 5µg/kg	21% oxygen	0,838442036	0,856976607	0,911897189	1,130428939	0,826009815	1,151041255	0,937342093	1,125292257	1,112069688	0,900073455
10	DEX 5µg/kg	21% oxygen	0,840993571	0,876057237	0,787973951	1,039254646	0,508210254	1,163757322	0,870371471	0,863473303	1,216266508	1,142014093
11	DEX 5µg/kg	21% oxygen	1,12424471	1,206874435	1,140842449	1,006752638	0,438087228	1,116289562	0,909962162	0,970544854	1,214015152	1,321590375
12	DEX 5µg/kg	21% oxygen	0,943514146	1,128468681	1,064389323	1,044104135	0,603176822	1,023942976	0,857129397	0,936385892	0,866432632	0,974138986
13	NaCl	80% oxygen, 24h	0,697199887	1,038499359	0,803229142	1,465827593	0,808799502	1,323581572	0,881793927	1,009478662	1,078342527	1,105807121
14	NaCl	80% oxygen, 24h	0,758810916	1,092199521	0,798366838	1,447785521	0,696446316	0,999922574	0,817864175	0,855227	0,797511018	0,92562653
15	NaCl	80% oxygen, 24h	0,758546517	1,357184025	0,928747588	1,402207862	0,754367159	1,206062932	0,87720722	0,929595602	1,024066038	1,168285872
16	NaCl	80% oxygen, 24h	0,772881366	1,507576797	0,852346023	1,446163929	0,612700944	0,991190965	0,95130383	0,781668463	0,754699276	1,030288845
17	NaCl	80% oxygen, 24h	0,522001876	1,361789407	0,610648887	2,174073867	0,724356456	1,403155588	1,111586738	1,265127305	1,272256199	1,276238143
18	NaCl	80% oxygen, 24h	0,633680486	1,328638011	0,660253348	1,820298106	0,79050073	1,194653294	1,039874033	1,136834755	1,270959018	1,161071361
19	DEX 5µg/kg	80% oxygen, 24h	1,001233355	1,118574149	1,147712036	1,735979458	0,71202292	1,140024016	0,902935626	0,897787798	1,240855936	1,039324568
20	DEX 5µg/kg	80% oxygen, 24h	0,809276176	1,161799599	0,822069303	1,677046785	0,890224389	1,354684899	1,078658275	1,207893233	1,393414736	0,956652549
21	DEX 5µg/kg	80% oxygen, 24h	0,767075515	1,404034117	0,926224551	1,961556733	0,7214628	1,181584013	1,213456913	1,183856002	1,318703018	1,288679091
22	DEX 5µg/kg	80% oxygen, 24h	1,261846501	1,617729178	1,168876747	1,90082006	0,352953603	0,997046144	1,214074182	1,276497636	1,396802594	1,159959003
23	DEX 5µg/kg	80% oxygen, 24h	1,145460156	1,494133657	1,183162446	1,340958322	0,463627813	0,966761861	1,13299563	1,07006452	1,438641278	1,111459707
24	DEX 5µg/kg	80% oxygen, 24h	1,206316304	1,199460849	0,980175705	1,51704234	0,709774307	1,075647292	1,151958752	1,133543995	1,527084023	1,057675179

Supplementary Table S3. Underlying $2^{-\Delta\Delta Ct}$ values utilized for the generation of RT-qPCR box-and-whisker plots at P11

sample	treatment		Syp 2^(-ddCT)	Tbr1 2^(-ddCT)	Tbr2 2^(-ddCT)	TNFa 2^(-ddCT)
1	NaCl	21% oxygen	1,204541354	1,193656851	0,963480805	1,193527541
2	NaCl	21% oxygen	0,778592507	0,735807885	0,933891745	1,041385873
3	NaCl	21% oxygen	1,042944875	0,924616689	0,918731977	1,012190508
4	NaCl	21% oxygen	0,777464944	1,062203124	1,048465853	0,980273951
5	NaCl	21% oxygen	1,119660981	1,189156823	1,117850433	0,964880455
6	NaCl	21% oxygen	1,174463033	0,974872081	1,032128034	1,003009592
7	DEX **5**µg/kg	21% oxygen	1,165767406	1,255366391	1,153291497	0,73441842
8	DEX **5**µg/kg	21% oxygen	0,815079653	0,755838932	0,682177748	0,837267071
9	DEX **5**µg/kg	21% oxygen	1,210785613	1,216339852	0,951324503	1,191859782
10	DEX **5**µg/kg	21% oxygen	1,050096094	0,819766438	1,134446153	1,406838221
11	DEX **5**µg/kg	21% oxygen	0,950258912	0,872590034	1,05424927	1,446665496
12	DEX **5**µg/kg	21% oxygen	0,896873594	0,797343051	0,985442488	1,267644082
13	NaCl	80% oxygen, 24h	1,228012602	1,168299688	0,467713335	1,241029404
14	NaCl	80% oxygen, 24h	1,038391312	1,0721583	0,710686317	1,110202585
15	NaCl	80% oxygen, 24h	1,088086154	0,949738746	0,483782932	0,879696697
16	NaCl	80% oxygen, 24h	0,959839361	0,951663892	0,50323534	0,755187675
17	NaCl	80% oxygen, 24h	1,203010775	1,223157358	0,84098707	1,145500056
18	NaCl	80% oxygen, 24h	1,267057367	1,251141868	0,745305302	1,153234525
19	DEX **5**µg/kg	80% oxygen, 24h	1,139756872	1,026106138	0,715195883	1,149760999
20	DEX **5**µg/kg	80% oxygen, 24h	1,195879236	1,242037897	0,897616485	1,064099214
21	DEX **5**µg/kg	80% oxygen, 24h	1,061696975	0,811379188	0,921042357	1,437527397
22	DEX **5**µg/kg	80% oxygen, 24h	1,035267342	1,008965726	1,217050884	2,620561647
23	DEX **5**µg/kg	80% oxygen, 24h	0,926607066	0,932666881	0,759831628	0,878155337
24	DEX **5**µg/kg	80% oxygen, 24h	1,053930168	1,086106878	0,764093517	1,03937715
