# Supplementary material for: Dexmedetomidine Preserves Hippocampal Neurogenesis During Recovery from Neonatal Hyperoxia in Rats
Source: Cells. 2026 Jun 16;15(12):1094. doi: 10.3390/cells15121094 (PMC13297234; doi:10.3390/cells15121094)
Supplement: Supplementary file 1 [file cells-15-01094-s001.zip › Table S4 2^-ddCt data P14.pdf]

Supplementary Table S4. Underlying $2^{-\Delta\Delta Ct}$ values utilized for the generation of RT-qPCR box-and-whisker plots at P14

			**Ascl1**	**Atg5**	**Atg12**	**AIF**	**BDNF**	**Beclin1**	**Casp3**	**Calb1**	**CycD2**	**Gclc**
**sample**	**treatment**		**2^(-ddCT)**	**2^(-ddCT)**	**2^(-ddCT)**	**2^(-ddCT)**	**2^(-ddCT)**	**2^(-ddCT)**	**2^(-ddCT)**	**2^(-ddCT)**	**2^(-ddCT)**	**2^(-ddCT)**
1	NaCl	21% oxygen	1,089127332	1,166334862	1,013103878	1,176035703	0,954403676	1,200907454	1,064786572	1,221067027	1,1279972	1,039702504
2	NaCl	21% oxygen	0,853379695	0,974760194	1,111216151	0,865242144	0,908484871	0,890427971	0,994888093	0,981661992	0,859583839	0,880453763
3	NaCl	21% oxygen	0,940988912	1,013039983	1,167762127	0,832404855	1,149563323	0,901798039	1,002366354	0,817453201	0,927821647	0,878968945
4	NaCl	21% oxygen	1,034298346	1,059915886	1,023651244	0,980317152	1,045896995	1,005618968	1,026283535	0,901983306	1,120962223	1,064716047
5	NaCl	21% oxygen	0,921740181	0,90678259	0,880632536	1,062334186	1,160749784	0,989409673	1,035083405	0,97913319	1,023952287	1,30725801
6	NaCl	21% oxygen	1,199334099	0,903395236	0,84381326	1,133651236	0,826398978	1,04225208	0,886531177	1,15556758	0,968430677	0,892926503
7	DEX **5**µg/kg	21% oxygen	0,750650194	0,908763625	1,017341193	1,078994245	1,306363196	0,965002357	1,018553131	1,383454257	1,19807005	0,786794671
8	DEX **5**µg/kg	21% oxygen	1,232296578	1,044849002	0,932792524	0,97368876	1,667456117	0,993362695	0,99624019	1,315471657	1,172504834	0,794325823
9	DEX **5**µg/kg	21% oxygen	1,032358524	1,003913741	0,876045693	1,068854062	1,39254281	1,060498085	1,407150913	1,273514745	1,220444983	0,668846785
10	DEX **5**µg/kg	21% oxygen	0,956952682	0,888657605	0,83127713	1,100823265	1,265464043	1,010867309	1,14774326	1,327242867	1,146637422	1,1827206
11	DEX **5**µg/kg	21% oxygen	0,887541873	1,024008536	1,072534784	1,210029496	1,319099551	1,035840605	1,10286087	1,34989145	1,132734326	1,069602798
12	DEX **5**µg/kg	21% oxygen	0,995067562	0,8057363	0,968510816	0,956655921	1,392451989	1,049331494	1,241224393	1,397096123	1,073195871	0,822049898
13	NaCl	80% oxygen, 24h	0,7559198	0,900344642	1,003716937	1,098736306	1,003305537	0,909058462	1,305042779	1,471701876	1,376674095	0,718717454
14	NaCl	80% oxygen, 24h	0,561300479	0,801672739	0,884606171	1,032667574	1,016889702	0,887786312	1,298314592	1,146414534	1,229802287	0,812811961
15	NaCl	80% oxygen, 24h	0,737888955	0,873568301	0,811822216	0,964821777	0,879463515	0,836036579	1,257090587	1,136564986	1,196698407	0,710049554
16	NaCl	80% oxygen, 24h	0,707045956	0,945586105	0,881896666	1,039779483	1,005049999	0,922281022	1,486692585	1,297834813	1,479669423	0,93882277
17	NaCl	80% oxygen, 24h	0,679748883	0,9207163	0,91495815	0,990621835	1,175617863	0,979358399	1,560886993	1,49444461	1,665414752	0,935200941
18	NaCl	80% oxygen, 24h	0,660365739	0,820430186	0,886299687	0,917024977	0,958006565	0,821144962	1,237472318	1,024071814	1,267944516	0,9939943
19	DEX **5**µg/kg	80% oxygen, 24h	1,256093415	0,80518221	0,956147071	0,977373496	1,339210432	0,791751053	1,054236011	1,189857563	0,910205048	0,903539333
20	DEX **5**µg/kg	80% oxygen, 24h	0,871936082	0,840733594	1,017642966	1,068802249	1,114100779	0,901316104	1,184939351	1,268189878	0,887118039	0,823871233
21	DEX **5**µg/kg	80% oxygen, 24h	1,095735349	0,894283387	1,008322386	1,007245968	1,206001882	0,884076916	1,254252609	1,044203742	0,863671047	0,887062553
22	DEX **5**µg/kg	80% oxygen, 24h	1,208203857	0,903969306	1,055014398	1,115402309	1,419834054	0,799719339	1,095985075	1,298978634	0,8650431	0,810652924
23	DEX **5**µg/kg	80% oxygen, 24h	1,13446876	0,939669473	0,944313125	1,076076705	1,158677987	1,030958165	1,278529218	1,102183808	1,081473096	1,037701694
24	DEX **5**µg/kg	80% oxygen, 24h	1,238478503	0,839016213	0,931402078	0,966418829	1,277250594	0,749908521	0,835495611	1,063272702	0,923045556	0,687536221

Supplementary Table S4. Underlying $2^{-\Delta\Delta Ct}$ values utilized for the generation of RT-qPCR box-and-whisker plots at P14

sample	treatment		GFAP 2^(-ddCT)	Hes5 2^(-ddCT)	Keap1 2^(-ddCT)	NeuN 2^(-ddCT)	NeuroD1 2^(-ddCT)	NeuroD2 2^(-ddCT)	NGF 2^(-ddCT)	Nrf2 2^(-ddCT)	Nrg1 2^(-ddCT)	Nrp1 2^(-ddCT)
1	NaCl	21% oxygen	1,111228352	1,002336582	0,9635931	1,180596042	1,078696034	0,999121795	1,140631695	1,119519426	1,044137863	1,014659859
2	NaCl	21% oxygen	0,959440188	0,870415416	0,802805073	0,839320637	0,857580235	0,966858592	0,831981466	0,953913371	1,070068435	0,858907928
3	NaCl	21% oxygen	0,97531064	0,84584286	1,005730992	0,841280407	0,930670131	1,1653981	0,860446597	0,934520919	1,251346332	1,210242886
4	NaCl	21% oxygen	1,054969912	1,003776742	1,082602884	1,041636004	1,166119099	0,860476175	1,14386439	0,980893805	1,037780186	1,048381844
5	NaCl	21% oxygen	1,062290983	1,0792705	1,246045404	0,916763613	1,122830811	1,092882603	0,958609678	1,189906958	0,85129053	1,227702999
6	NaCl	21% oxygen	0,858128349	1,250842769	0,95282102	1,256193815	0,887101164	0,944565193	1,116864902	0,858490327	0,809598884	0,736626881
7	DEX **5**µg/kg	21% oxygen	1,024637366	1,037592358	1,013067149	1,112295621	0,78368915	1,165178822	1,416701527	0,987122207	1,081722623	1,026799182
8	DEX **5**µg/kg	21% oxygen	1,378175911	1,307180757	0,987987848	1,099472525	1,332532941	1,019761764	1,218804064	1,12321141	0,923244777	1,164223004
9	DEX **5**µg/kg	21% oxygen	0,681519429	0,777559582	0,680456101	1,762571288	0,851274073	1,053713211	1,478805021	0,846395651	1,179544231	1,248646172
10	DEX **5**µg/kg	21% oxygen	1,273467725	1,071916349	1,189668196	1,215507647	0,878754287	1,068943719	1,227753425	1,346116599	0,903509324	1,180391573
11	DEX **5**µg/kg	21% oxygen	1,149721791	1,118783325	1,235995393	1,176590882	0,790624189	0,935276914	1,175366049	1,214269199	1,174889662	1,123676169
12	DEX **5**µg/kg	21% oxygen	0,720600235	1,067148819	1,007375339	1,257818998	1,20896963	1,135430238	1,20234669	0,897838073	1,194187632	1,168714274
13	NaCl	80% oxygen, 24h	1,093865694	0,801381652	0,981209861	1,177166573	0,687181742	1,390203277	1,090760321	1,031532027	1,041981106	1,063562008
14	NaCl	80% oxygen, 24h	1,016155109	0,576365215	0,934318332	0,972656888	0,673002945	0,915715713	0,965754042	1,190451338	0,872693328	0,83483284
15	NaCl	80% oxygen, 24h	0,97778734	0,758711585	1,229069934	1,219683873	0,786718798	1,382100179	1,193567796	0,960855885	1,025388122	0,872965467
16	NaCl	80% oxygen, 24h	1,133632959	0,81464119	1,283599769	1,2566721	0,89345914	1,593649651	0,985163507	1,279476198	1,206613338	1,139527168
17	NaCl	80% oxygen, 24h	1,066622099	0,80874636	1,156873742	1,267709537	0,784363385	1,422985672	1,244030607	1,14605619	1,222137283	1,299456907
18	NaCl	80% oxygen, 24h	1,093526861	0,802205083	1,19733401	1,101408462	0,704807304	1,003105839	1,062406535	1,191474527	1,031970448	1,003788789
19	DEX **5**µg/kg	80% oxygen, 24h	1,29513223	0,841568871	1,139745788	0,963420086	1,243601981	1,228748675	1,073674412	1,135367026	1,07156341	1,353800338
20	DEX **5**µg/kg	80% oxygen, 24h	0,969667283	0,873055788	1,006232839	0,976913581	0,914323658	0,989902096	1,064691899	0,989542728	0,975163663	1,001590896
21	DEX **5**µg/kg	80% oxygen, 24h	1,212047188	0,750717714	1,114204772	0,946272004	0,831796415	0,902878089	1,060935087	1,309317333	1,219992045	1,101963474
22	DEX **5**µg/kg	80% oxygen, 24h	1,253709781	0,721542194	1,049399366	1,032801363	1,226099563	1,357632154	0,954194283	1,135948076	1,169865703	1,238766894
23	DEX **5**µg/kg	80% oxygen, 24h	1,188523929	0,98167112	0,77660877	1,300302446	1,101193848	1,374605995	1,194445899	1,128697067	0,96057994	1,298301795
24	DEX **5**µg/kg	80% oxygen, 24h	0,921390513	0,886488437	0,720671314	0,997171583	1,462571248	1,162293745	1,195094156	1,258215854	0,741377208	1,44860932

Supplementary Table S4. Underlying $2^{-\Delta\Delta Ct}$ values utilized for the generation of RT-qPCR box-and-whisker plots at P14

			**NT3**	**Pax6**	**Prox1**	**Scl1a3**	**Sema3a**	**Sema3f**	**SOD1**	**SOD2**	**SOD3**	**Sox2**
**sample**	**treatment**		**2^(-ddCT)**	**2^(-ddCT)**	**2^(-ddCT)**	**2^(-ddCT)**	**2^(-ddCT)**	**2^(-ddCT)**	**2^(-ddCT)**	**2^(-ddCT)**	**2^(-ddCT)**	**2^(-ddCT)**
1	NaCl	21% oxygen	0,847967942	1,172859662	0,733500417	1,00765478	1,26854163	1,171438583	0,90464905	1,099103006	1,220767693	1,071143855
2	NaCl	21% oxygen	1,015181205	0,868792464	1,11882668	0,842588607	0,924496031	0,74451465	0,976194703	0,971505482	0,802651202	1,136101371
3	NaCl	21% oxygen	1,206081885	1,080704049	0,965855042	1,091662113	0,920303466	1,000716938	0,912230189	0,985994333	0,850100704	0,989666234
4	NaCl	21% oxygen	1,121605013	1,069412635	1,130845436	1,073099804	1,228542201	0,977523944	1,058004139	1,148517851	1,110425447	1,00335752
5	NaCl	21% oxygen	0,858737169	0,786781195	1,080289261	1,248491263	0,825734556	0,963764177	1,169645798	1,866875853	1,298732199	0,996204059
6	NaCl	21% oxygen	0,118297648	1,079274321	1,032717559	0,805302017	0,913331903	1,216179822	1,003083876	0,826997469	0,832454917	0,830696433
7	DEX **5**µg/kg	21% oxygen	0,743850507	0,794568773	0,75891916	1,171320025	0,760549765	1,131509653	1,06677508	0,978478572	0,774014461	1,06602783
8	DEX **5**µg/kg	21% oxygen	1,071476773	0,869037535	1,175081768	1,335630581	0,924761298	1,165969308	1,106855037	1,270867164	1,149207558	1,172820105
9	DEX **5**µg/kg	21% oxygen	0,783662693	0,837780449	0,766913566	1,16120993	1,298092843	1,299242368	0,912028803	0,956484012	0,961507385	0,978954222
10	DEX **5**µg/kg	21% oxygen	0,750143029	0,776792063	1,112846163	1,584457126	0,976630313	1,142413395	1,23531614	1,616498863	1,296168476	1,107677961
11	DEX **5**µg/kg	21% oxygen	0,701730811	1,02234819	0,715547271	1,40112853	0,874682862	1,030061518	1,00774131	1,313463545	1,117019428	1,095645633
12	DEX **5**µg/kg	21% oxygen	0,920111351	0,667281388	0,84815222	1,166826866	1,307220622	1,291838983	0,912149388	0,873692893	0,745036514	0,885766426
13	NaCl	80% oxygen, 24h	0,463026197	0,797982084	0,932217664	1,331237797	0,920609316	1,012386468	0,894641093	0,606721202	0,709756248	0,988346056
14	NaCl	80% oxygen, 24h	0,406309325	0,606416397	0,725752663	1,186409973	0,928792829	0,896178142	0,856660845	1,06927393	0,807880599	0,854573972
15	NaCl	80% oxygen, 24h	0,507835155	0,752614808	1,05624819	1,311294835	0,931055385	1,086959299	0,969862347	1,041006499	0,912471997	1,053161539
16	NaCl	80% oxygen, 24h	0,582878427	0,753637113	0,879339656	1,989755054	1,151434139	1,179985654	1,104720373	1,471451811	1,026928394	1,012701826
17	NaCl	80% oxygen, 24h	0,672100798	0,829189964	1,161224788	1,313103677	0,699671702	1,157501828	1,05124024	0,965198653	0,913909495	1,248364943
18	NaCl	80% oxygen, 24h	0,685400053	0,757955055	0,889737674	1,495985579	0,863036406	1,019204181	1,071497025	1,290013915	0,858955066	0,97017353
19	DEX **5**µg/kg	80% oxygen, 24h	0,779882487	0,648631536	1,376096104	1,476720196	0,582092716	1,257207909	1,189451908	1,085869579	0,864045092	1,011337548
20	DEX **5**µg/kg	80% oxygen, 24h	0,636520904	0,827730606	1,209540943	1,166860496	0,644215157	0,969963741	0,93384849	0,922787804	0,66903117	0,978372415
21	DEX **5**µg/kg	80% oxygen, 24h	0,964784684	1,003376062	1,399564521	1,460482126	0,665690915	1,099766818	1,181985421	1,214695442	0,934177539	1,048789357
22	DEX **5**µg/kg	80% oxygen, 24h	0,833658813	0,78724449	1,375192936	1,183405778	0,706121174	1,033719938	1,258240944	1,109505711	0,797358664	1,069237082
23	DEX **5**µg/kg	80% oxygen, 24h	0,673398705	0,672756576	1,223600524	1,759433867	1,128431334	1,292667583	1,228265113	1,208837312	0,849093317	1,031928321
24	DEX **5**µg/kg	80% oxygen, 24h	0,800071879	0,752401905	1,386716225	1,233349047	1,121754167	0,856367076	1,261024815	1,074454303	0,872980983	0,883048309

Supplementary Table S4. Underlying $2^{-\Delta\Delta Ct}$ values utilized for the generation of RT-qPCR box-and-whisker plots at P14

			Syp	Tbr1	Tbr2	TNFa
**sample**	**treatment**		**2^(-ddCT)**	**2^(-ddCT)**	**2^(-ddCT)**	**2^(-ddCT)**
1	NaCl	21% oxygen	1,173074854	1,239420543	1,080778472	0,903025401
2	NaCl	21% oxygen	0,846635759	0,795660631	1,151929615	0,967647855
3	NaCl	21% oxygen	0,878251097	0,906413578	0,830641482	1,038981537
4	NaCl	21% oxygen	1,033240247	1,10226015	1,168142918	0,835516611
5	NaCl	21% oxygen	1,020106377	0,874389983	0,886060228	1,095744974
6	NaCl	21% oxygen	1,087707663	1,160747499	0,934253066	1,203123721
7	DEX **5**µg/kg	21% oxygen	1,134545871	1,047925398	1,360912269	0,977259333
8	DEX **5**µg/kg	21% oxygen	1,201367107	0,886858503	1,223437998	0,822837101
9	DEX **5**µg/kg	21% oxygen	1,277535777	1,240347247	1,176916801	0,785421674
10	DEX **5**µg/kg	21% oxygen	1,305470471	1,048255183	0,758522703	1,000285138
11	DEX **5**µg/kg	21% oxygen	1,151298833	1,026225947	1,050057533	3,645631986
12	DEX **5**µg/kg	21% oxygen	1,219220668	1,189606508	1,008806034	1,169602441
13	NaCl	80% oxygen, 24h	1,131498567	1,068677423	0,721027342	1,098271537
14	NaCl	80% oxygen, 24h	0,934228823	0,842689303	0,7602383	2,424419492
15	NaCl	80% oxygen, 24h	1,06889129	0,986854085	0,637227625	0,820666443
16	NaCl	80% oxygen, 24h	1,290552299	1,257029354	0,595778147	1,150312275
17	NaCl	80% oxygen, 24h	1,260621971	1,161533477	0,70352928	0,833603771
18	NaCl	80% oxygen, 24h	0,98376683	0,863130379	0,541661949	1,045975953
19	DEX **5**µg/kg	80% oxygen, 24h	1,152382572	0,678310177	1,109602032	0,991767123
20	DEX **5**µg/kg	80% oxygen, 24h	0,91850698	0,837764217	0,865284636	1,078281194
21	DEX **5**µg/kg	80% oxygen, 24h	0,902299066	0,781154894	0,820707118	1,189426163
22	DEX **5**µg/kg	80% oxygen, 24h	1,020269578	0,753892835	0,879548516	3,21519722
23	DEX **5**µg/kg	80% oxygen, 24h	1,200649941	0,909362459	0,962218023	1,257975304
24	DEX **5**µg/kg	80% oxygen, 24h	0,968042003	0,711048123	0,722836377	0,763735224
